# Patient engagement in research: a systematic review

**DOI:** 10.1186/1472-6963-14-89

**Published:** 2014-02-26

**Authors:** Juan Pablo Domecq, Gabriela Prutsky, Tarig Elraiyah, Zhen Wang, Mohammed Nabhan, Nathan Shippee, Juan Pablo Brito, Kasey Boehmer, Rim Hasan, Belal Firwana, Patricia Erwin, David Eton, Jeff Sloan, Victor Montori, Noor Asi, Abd Moain Abu Dabrh, Mohammad Hassan Murad

**Affiliations:** 1 Knowledge and Evaluation Research Unit, Mayo Clinic, Rochester, MN, USA; 2 Unidad de Conocimiento y Evidencia, Lima, Perú; 3 Division of Preventive, Occupational and Aerospace Medicine, Mayo Clinic, Rochester, MN, USA; 4 Division of Endocrinology, Diabetes, Metabolism, Nutrition, Mayo Clinic, Rochester, MN, USA; 5 Center for the Science of Healthcare Delivery, Mayo Clinic, Rochester, Minnesota, USA; 6 Department of Health Sciences Research, Mayo Clinic, Rochester, MN, USA; 7 Mayo Clinic Libraries, Mayo Clinic, Rochester, MN, USA; 8 Department of Internal Medicine, University of Missouri, Columbia, MO, USA

**Keywords:** Systematic review, Patient, Engagement, Patient centered outcomes research

## Abstract

**Background:**

A compelling ethical rationale supports patient engagement in healthcare research. It is also assumed that patient engagement will lead to research findings that are more pertinent to patients’ concerns and dilemmas. However; it is unclear how to best conduct this process. In this systematic review we aimed to answer 4 key questions: what are the best ways to identify patient representatives? How to engage them in designing and conducting research? What are the observed benefits of patient engagement? What are the harms and barriers of patient engagement?

**Methods:**

We searched MEDLINE, EMBASE, PsycInfo, Cochrane, EBSCO, CINAHL, SCOPUS, Web of Science, Business Search Premier, Academic Search Premier and Google Scholar. Included studies were published in English, of any size or design that described engaging patients or their surrogates in research design. We conducted an environmental scan of the grey literature and consulted with experts and patients. Data were analyzed using a non-quantitative, meta-narrative approach.

**Results:**

We included 142 studies that described a spectrum of engagement. In general, engagement was feasible in most settings and most commonly done in the beginning of research (agenda setting and protocol development) and less commonly during the execution and translation of research. We found no comparative analytic studies to recommend a particular method. Patient engagement increased study enrollment rates and aided researchers in securing funding, designing study protocols and choosing relevant outcomes. The most commonly cited challenges were related to logistics (extra time and funding needed for engagement) and to an overarching worry of a tokenistic engagement.

**Conclusions:**

Patient engagement in healthcare research is likely feasible in many settings. However, this engagement comes at a cost and can become tokenistic. Research dedicated to identifying the best methods to achieve engagement is lacking and clearly needed.

## Background

The role of patient in research ranges from a passive one (patient is a data point) to an active one (patient is a researcher). The active participation in research (or patient engagement in research) can potentially lead to improvement in the credibility of results (higher rates of enrollment and retention) and in their direct applicability to patients (by asking pertinent questions about patient-important outcomes). Also, there is an overarching ethical mandate for patient participation in research as a manifestation of the “democratization” of the research process [1-3]. Patient engagement in the planning and execution of research could also improve its translation into clinical practice [4]. In all, there is growing consensus about the crucial role of patient involvement in research, which may improve the value of healthcare research.

In the United Kingdom, involvement of stakeholders in social and health care policy has been well recognized since 1996. The British National Institute of Health recognized that individual and community stakeholders determine important aspects of health care services and research, and the project INVOLVE was established to achieve this engagement [5,6]. In the United States, the Patient Centered Outcomes Research Institute (PCORI) was established in 2010 and placed great importance on the engagement of patients and other stakeholders in the research process [7].

Previous systematic reviews have described various aspects of the engagement process [8,9]. However, it remains unclear who to engage or when, or how to perform this task [8-10]. Therefore, PCORI commissioned a systematic review that aims at synthesizing the existing evidence about patient engagement in research with the goal of helping researchers in designing and conducting meaningful patient engagement in healthcare research. In this systematic review we aimed to answer 4 key questions: what are the best ways to identify patient representatives? How to engage them in designing and conducting research? What are the observed benefits of patient engagement? What are the harms and barriers of patient engagement?

## Methods

This systematic review is conducted based on a priori established protocol (Additional file 1) and is reported according to the PRISMA statement [11]. The PRISMA checklist is available in Additional file 2.

### Eligibility criteria

We included all original studies of any design, size, or patient population published in the English language in which patients or their surrogates provided feedback, had input, or took part in the design, conduct and dissemination of research. Systematic reviews were also included to supplement the findings from original studies. Other non-original studies (non-systematic literature reviews, comments, opinions, letters and editorials etc.) were excluded. In general, we sought studies in which patients were actively engaged in designing research. Participation in surveys was only considered to be research engagement when the main purpose of the survey was to obtain patients’ values and preferences that relate to research prioritization or research design.

### Patient advisory group

The protocol of this systematic review was developed after consultation with patients from the Patient Advisory Council [12]. This is a group of volunteer patients from Rochester, Minnesota who have contributed to the design of multiple studies over the last 10 years. The group helped in developing the questions and outcomes of the review and advised on terminology. They also reviewed the results and provided feedback on the presentation of findings, usefulness and applicability.

### Search

#### Electronic search

An expert librarian (PJE) collaborated with a methodologist (MHM) to develop the search strategy. We searched biomedical electronic databases [PubMed/Ovid MEDLINE, Ovid EMBASE, Ovid PsycInfo, Ovid Cochrane, EBSCO CINAHL, SCOPUS, Web of Science (multidisciplinary scientific content), Business Search Premier, Academic Search Premier and Google Scholar (communications, marketing, public opinion, and business literature that incorporate non-healthcare resources)] from their inception through November 2011 (Additional file 1).

To identify additional candidate studies we reviewed reference lists from eligible studies and conducted additional MEDLINE searches using the PubMed “related articles” feature for eligible studies. We used SciSearch for publications that cited eligible studies to supplement the database search.

#### Environmental scan

We complemented the database search with an environmental scan and a manual search. The environmental scan includes searching the Internet using various search engines for recent and ongoing activities, initiatives, white papers and websites to identify key players and trends in the field. It helps provide content from grey (unpublished) literature and from fields other than medicine. We also searched the scientific search engines Scirus and Sciverse, which contain scientific journal content, scientists’ homepages, courseware, pre-print server material, patents, and institutional repository and website information. In addition, we contacted experts in the field to identify other relevant documents (e.g., dissertations, scientific reports). The environmental scan, as expected, identified some of the published literature already included in the systematic review; overlapping references were excluded.

### Study selection

We collated initial references in citation files (using Endnote software), removed duplicates, and screened titles and abstracts against eligibility criteria using DistillerSR software (Evidence Partners Incorporated, Ottawa, Canada). Studies were reviewed in duplicate until almost perfect agreement (Kappa > 0.80) [13] was achieved, after reviewing 200 potentially includible references. Disagreements in the initial screening were automatically included. Potentially eligible studies were then reviewed in full text following a similar procedure. Disagreements in full-text screening were reconciled by discussion, consensus, or arbitration by study principal investigator (MHM). We exclusively used electronic file formats (Portable Document Format/PDF), in reference management software to reduce costs and paper use.

### Data extraction

Data were extracted from included studies using a standardized form developed based on the protocol. We tested this form using a small sample (n = 10) of randomly selected studies, from which all reviewers extracted data. The first author (JPD) evaluated each extraction form and compared the extracted data between reviewers and discussed discrepancies with them.

Data extracted from each study included: study description (e.g., demographics of participants and research setting), methods used to select patients (defined as a patient, surrogate, caregiver, community member, or other stakeholder informing research), measures set in place to enhance the validity or completeness of identifying patients (e.g., selecting methods –convenience, random, volunteer, training level for the task), measures of validity or accuracy of the information or input given by participants (the patient’s voice, e.g., validation of the patient reported outcome measurement, congruence of patient’s voice with other stakeholders), description of methods used to implement/incorporate the patient’s voice in research, and any reported outcomes of patient engagement. We also captured authors’ recommendations about the methods to be used for eliciting the patient’s voice and facilitating patient engagement.

### Analysis

The nature of the question of this systematic review along with the lack of standard approach across existing studies prevented a quantitative meta-analysis. Instead, data extracted from the included studies were analyzed using a meta-narrative approach [14]. This approach was developed as a pragmatic solution to study topics that have been differently conceptualized and studied by different groups of researchers [15]. The approach starts by a standard systematic review with explicit inclusion and exclusion criteria. Analysis follows a framework that defines the key questions. The included studies are evaluated until saturation for discrete themes and trends that can be mapped to outcomes. Differences in studies settings and characteristics can be used to explain differences in results (heterogeneity) [16].

**Figure 1:**
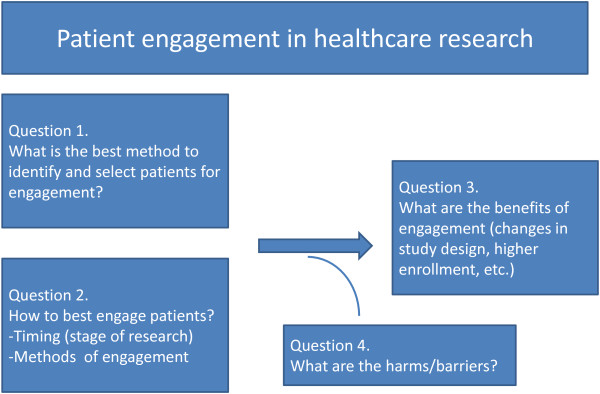
Analytical framework.

The analytic framework of this systematic review is depicted in Figure 1 showing the 4 key questions of interest. Following this approach, patient engagement experiences reported in the literature were classified into categories based on how patients were selected and engaged. Then, we attempted to map each category to an engagement outcome corresponding to the 4 key questions to allow inference.

We also categorized the research engagement from each study into three different research phases proposed for patient engagement [17]:

1) Preparatory phase (agenda setting, prioritization of research topics and funding).

2) Execution phase (study design & procedures, study recruitment, data collection, and data analysis).

3) Translation phase (dissemination, implementation, and evaluation).

Results are presented following the sequence of the 4 key questions as depicted in the framework (Figure 1) with illustrative examples.

## Results

### Search and selection results

Overall we identified 5,562 possibly relevant citations, of which 142 met the eligibility criteria and were included. Studies reported a spectrum of engagement. Studies described patient engagement in research preparation phase (35), execution phase (90) and translation phase (52). Some studies contributed to our understanding of more than one phase.

In terms of the 4 key questions of this review, we found 121 studies that contributed to our understanding of the first question regarding patient selection; 45 studies that contributed to our understanding of the second question regarding engagement methods; 43 studies that reported on the third question regarding engagement outcomes; and 36 studies that reported on the fourth question regarding barriers and challenges of engagement. Many studies contributed to our understanding of more than one of the 4 key questions.

**Figure 2:**
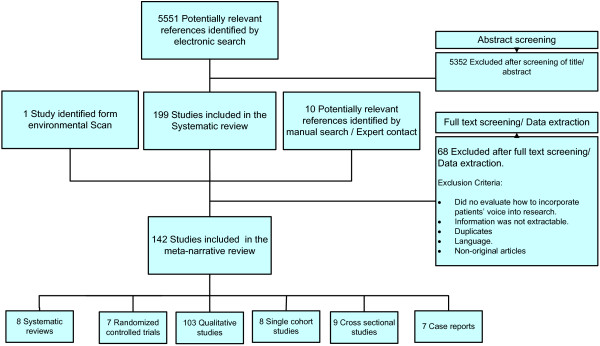
Study selection process.

The study selection process is described in Figure 2. The studies included 8 systematic reviews, 7 randomized trials and 24 observational studies; the remaining majority (103) consisted of qualitative studies. Additional file 3: Tables S1 and S2 describe the characteristics of the systematic reviews and original studies. Due to the qualitative component of our research question and the heterogeneity of studies design, the methodological quality of the included studies was evaluated using selected items from the list proposed by the Critical Appraisal Skills Programme (CASP) [18,19]. The studies overall had a few limitations particularly in the areas of patient selection and data synthesis (Additional file 3: Table S3). Additional file 3: Table S4 shows the list of initiatives and patient organizations engaging patients in research identified by the environmental scan.

### Overview of existing systematic reviews

The literature search identified 8 relevant systematic reviews that addressed various aspects of patient engagement and spanned across the 4 key questions. A Cochrane systematic review by Nilsen et al. [8] reported that engaging patients in the research process may lead to an output (report) that is more readable and understandable by other patients. The authors concluded that there are insufficient data to evaluate the impact of patient engagement. Nevertheless, they found that the engagement was feasible in most of their included randomized controlled trials.

The systematic review by Mockford et al. [9] evaluated the impact of patient and public involvement in the UK National Health Service programs. The review summarized 42 studies and concluded that there is little evidence of any economic analysis of the costs involved, poor quality of reporting, minimal theoretical or conceptual underpinning, lack of measurement and evaluation; and overall weak evidence base to support patient and public engagement.

Brett et al. [1] examined the conceptualization, measurement, impact and outcomes of patient and public involvement in health and social care research. They concluded that there is an emerging and important evidence of the impact of patient engagement on health care research but with relatively little conceptualization and theoretical development in the field. They also described poor quality of reporting as a one of the most important barriers restricting our understanding of the impact of patient engagement in research.

Boote et al. [20] reviewed published case examples of public involvement in primary research design and reported that group meetings were the most common method used to engage the public and that most patient contributions were in the areas of review of consent procedures and patient information sheets, outcome suggestion, and recommendations on participants recruitment.

Three systematic reviews by Legare et al., Oliver et al., and Stewart et al., summarized collectively over 250 studies in which patient engagement was conducted [10,21,22]. The three reviews reported similar key findings and focused on describing topics and stages of research most amenable to engagement and common methods of engagements (e.g., meetings, workshops and focus groups) that should be tailored to the topic of research at hand. These reviews also highlighted challenges and barriers to engagement [10,21,22].

Lastly, Hussain-Gambles [23] focused on engaging South Asian patients in designing clinical trials and reported on the factors that motivate patients to participate as well as deterrents. The review highlighted that there are more similarities than differences in attitudes towards clinical trials between the South Asian and the general population. The main findings and conclusions of the 8 systematic reviews are reported in Additional file 2: Table S3.

### Key questions

#### Question 1. What are the best methods to identify patients for engagement?

In general, most of the studies described convenience sampling as the method to identify patients (or representatives/surrogates) for engagement in research. Therefore, patients attending clinics or other patient care facilities were approached and asked to participate. Patients also volunteered in a response to advertisements or Internet postings. Very few studies randomly selected patient representatives. For example, researchers in the Netherland randomly selected patients with asthma and chronic obstructive pulmonary disease from the entire pool of the Netherlands Asthma Foundation and engaged these patients in consultation to define their health research priorities [24]. This engagement resulted in additional prioritization of other research topics that were not covered by current Dutch research programs. Researchers in Canada randomly selected from patients attending outpatient cancer therapy to evaluate their attitudes, motivations and barriers to participation in clinical trials [25]. Murad et al. asked a random sample of patients with diabetes about their preferences for future trials in diabetes in terms of design (pragmatic vs. explanatory trial design) and outcomes (surrogate vs. hard endpoints) [26].

We found no comparative analytic studies to provide evidence supporting a particular method to identify or select patients for engagement in research.

#### Question 2. What are the best methods to engage patients?

Studies described a variety of methods that were used to engage patients, the most common of which were focus groups, interviews, surveys and the most active form of engagement which is serving on a study board or advisory council and attending regular meetings with researchers (as in active participatory research studies and community based participatory research). For example, Swartz et al. conducted a randomized controlled trial to test the effectiveness of a pollutant and allergen control strategy on the symptoms of childhood asthma [27]. In the early stages of the study, researchers engaged several stakeholders (2 school principals, a pastor, a nun, 2 community association presidents, a social worker, a parent of a child with asthma and a health care worker who had previously served in the same community). The engaged persons took the role of an advisory board and a partner in research. They helped develop study protocol, recruitment procedures and selected outcomes. They also educated study personnel about the community and attended presentations and meetings to obtain necessary approvals for the study. They subsequently even helped with execution of study intervention (measured pollutant levels in demolition sites). Crowe et al. described the engagement of Hispanic farm workers and their families in Yakima Valley, Washington, in every stage of a study including study concept and design, data collection, data analysis and interpretation, conclusions, and dissemination of results [28]. Thirteen community members and stakeholders met regularly throughout the duration of the study providing input and making decisions about research execution. The engaged persons also presented alongside researchers to the larger community during Town Hall meetings.

**Figure 3:**
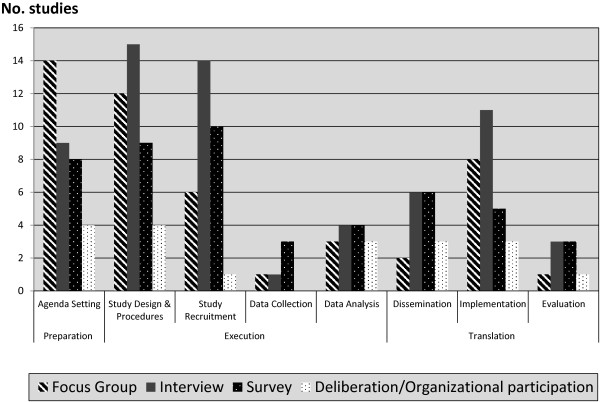
Methods and phases of engagement.

We found no comparative analytic studies to provide evidence supporting a particular method of engagement. The different methods used for engagement are depicted in Figure 3 and are stratified according to research phase.

#### Question 3. What are the observed benefits of patient engagement?

Several studies reported that engaging patients in research improves patient enrollment and decrease attrition [10,27,29-31]. For instance, Edwards et al. [30] conducted a randomized controlled trial of osteopathy in children with cerebral palsy compared to a control group that only received standard therapy. They demonstrated that engaging parents in study design led to higher enrollment and retention rates. Likewise, Swartz et al. [27] conducted a randomized controlled trial in inner-city children with asthma comparing environmental control education, allergen-proof encasements, pest extermination, and an air filter to a control group that only received standard therapy. The used a community based participatory research approach, which achieved a high enrollment and retention rates, 86% and 70% respectively. They also reported that engagement helped in dissemination to the extent that their reporting was more meaningful and understandable for participants and the community.

#### Question 4. What are the harms and barriers of patient engagement?

While most studies reported mainly positive effects of engagement, [1] a smaller number described potential harms or adverse effects of engaging patients. These harms mainly related to patient frustration with the lengthy process that involved training, transportation, attendance, etc. [31,32]. In terms of barriers and challenges, studies cited logistics such as extra time needed to complete research, time constraints of patients and researchers, and incremental funding needed for patient engagement. Another common concern and an overarching worry of researchers and patients was that patient engagement may become tokenistic [33,34] (a false appearance of inclusiveness), resulting in a devaluated patients’ input. Another potential challenge described was “scope creep”; a theoretical concern that engaging patients in the research may include irrelevant community concerns and issues, which would make the research unfeasible [27,35].

Of the few studies that described potential solutions, the most commonly described were spending adequate time to build reciprocal relationships [17] (between patients and researcher), fostering mutual respect and developing clear expectations that are explicitly described and documented in study protocols. We found no comparative studies to provide supportive evidence for question 4.

## Discussion

This meta-narrative systematic review identified numerous heterogeneous studies in which patients or their surrogates (other patients) were successfully engaged in the research process. The engagement was feasible in most of the published cases we found, but faced several challenges and barriers. Most studies claimed some benefits of this process; however, there were no comparative data to suggest best practices.

### Strengths and limitations

Heterogeneity of study populations, methods, and outcomes constitute limitations of this synthesis. Publication and reporting biases might have impacted the conclusions of this report and their impact cannot be estimated. Our search may have missed studies in which patient engagement was performed due to the lack of uniform reporting or indexing methods of engagement. Evaluating the quality of patient engagement is challenging due to unavailability of validated scales and the limited data reported in the studies. We did not find the standard tools for assessing the methodological quality of the studies particularly useful because such assessment actually relates to the outcomes of the study and not to the outcome of patient engagement. Such assessment can be misleading as it is quite possible to have a study with low risk of bias for its primary and secondary outcomes that performed tokenistic or ineffective patient engagement.

Our results are consistent with other systematic reviews in the field [1,8,9]. The current review updates the evidence base to date and provides a contemporary look at patient engagement. Developed with active participation from patients, researchers, and the PCORI staff (as an external experts’ advisory group that did not participate in conducting this SR), this systematic review takes priority in establishing the baseline starting point from which we need to advance the science of patient engagement in research. This review utilized a comprehensive and sensitive search strategy that spanned across multiple databases and was augmented by an environmental scan of non-peer-reviewed relevant sources to further capture related studies, web sites, and interest groups. Our application of an a priori protocol for selecting and appraising evidence reduces selection bias. The thematic analysis of this review sought to ensure presenting the evidence without over-interpreting its signals and silences, a key concern in this area.

### Practical implications

At the present time we are unable to recommend best practices on the basis of comparative evidence. However, many methods are described in the literature with reported success. In terms of identifying patients for engagement, random sampling is the least biased way although considering that the number of patients chosen for engagement is very small, random sampling can fail. This approach is also challenging in rare diseases. Most of the included studies used volunteers which is a more practical method despite the potential for having a sample of patients that are not truly representative of the targeted population. Volunteers may be more educated and motivated and engage more effectively, yet they may have personal agendas. At the present time, we suggest that researchers choose their method of selection based on the availability of subjects and the research topic at hand.

Engaging patients in all research phases (preparatory, execution and translation) seems feasible in most cases. This was even demonstrated in populations and communities with high prevalence of social inequities (intellectual disparities, poverty, unemployment and illiteracy) traditionally considered difficult to reach [36-40]. The engagement process may improve the credibility of results and their applicability to the target population and may have an empowering effect on participants. Potential risks (harms and costs) for engaged patients should be balanced against the broad range of articulated potential benefits.

Future research in this field is greatly needed to demonstrate the value of patient engagement to researchers and funders. For example, patient engagement when conducting systematic reviews has been recommended by the Agency for Healthcare Research and Quality Evidence-based Practice Centers, the Institute of Medicine, the Cochrane Collaboration, and others [41]. Yet, there is no guidance on how to perform this engagement. A possible study in this field can randomize systematic reviewers to test three approaches, one without engaging patients, one with engaging patients with the condition being studied, and one with engaging patients with any condition (general patients). Qualitative studies can be embedded in most trials to evaluate different engagement strategies or engagement in different phases of the trials. Clinical practice guidelines are mostly done without patient engagement;[42] however this engagement is described by many as paramount and essential [43,44]. The impact of patient representation on guideline panels cane be studied using qualitative research methods to determine if their presence was tokenistic or meaningful. Lastly, we recommend that bibliographic databases use indexing terms that identify active patient engagement in research to facilitate future research in this field.

## Conclusions

Patient engagement in healthcare research is likely feasible in many settings. However, this engagement comes at a cost and can become tokenistic. Research dedicated to identifying the best methods to achieve engagement is lacking and clearly needed.

## Competing interests

The authors declare that they have no competing interests.

## Authors’ contributions

All listed authors significantly contributed to this project. Study protocol was developed by MHM, VMM, JPD and PJE. JPD, GP, TE, ZW, NA, AA, MN, JB, KB, RH and BF carried out study selection and data extraction. NS, DE and JS provided the content expertise for study design and participated in writing the discussion section of the manuscript. PJE is the reference librarian who conducted the literature search. VMM and MHM provided the methodological expertise and supervised the work. All authors read and approved the final manuscript.

## Pre-publication history

The pre-publication history for this paper can be accessed here:

http://www.biomedcentral.com/1472-6963/14/89/prepub

## Supplementary Material

Additional file 1 Protocol and Search Strategy. Click here for file

Additional file 2 PRISMA 2009 checklist. Click here for file

Additional file 3: Table S1 Systematic reviews. **Table S2.** Original studies. **Table S3.** Quality assessment of the original studies. **Table S4.** Initiatives and patient organizations identified by the environmental scan. Click here for file
