# Supplementary material for: Patient engagement in research: a systematic review
Source: BMC Health Serv Res. 2014 Feb 26;14:89. doi: 10.1186/1472-6963-14-89 (PMC3938901; doi:10.1186/1472-6963-14-89)
Supplement: Additional file 1 — Protocol and Search Strategy. [file 1472-6963-14-89-S1.docx]

**Additional file 1: Protocol and Search Strategy**

**(1) Protocol**

**Eligibility criteria**

- Original studies of any design, size, or patient population published in the English language in which patients or their surrogates provided feedback, had input, or took part in the design, conduct and dissemination of research.
- Systematic reviews included.
- Non-original studies (non-systematic literature reviews, comments, opinions, letters and editorials etc.) are excluded.

**Patient Advisory Group**

- The protocol of this systematic review was developed after consultation with patients from the Patient Advisory Council
- The group helped in developing the questions and outcomes of the review and advised on terminology
- The group will review the results and provide feedback on the presentation of findings, usefulness and applicability.

**Environmental scan and Manual search:**

- Google
- Scientific search engines Scirus and Sciverse
- experts in the field

**Study selection**

- In Endnote, remove duplicates
- DistillerSR software (Evidence Partners Incorporated, Ottawa, Canada).
- in duplicate until almost perfect agreement (Kappa>0.80) is achieved after reviewing 200 potentially includible references
- Disagreements in the initial screening automatically included
- Potentially eligible studies reviewed in full text
- Disagreements in full-text screening reconciled by discussion, consensus, or arbitration by study principal investigator (MHM)

**Data extraction**

**Data extraction form**

Study Name

Primary goal of the study (text box)

Design

Demographic of the informants

- Age
- Sex
- Socio-economic status

Whose voice is being incorporated?

- Patients
- Relative
- Surrogate
- Other

Response rate

- No. Patients whose opinion was considered
- No. Patients invited
- Other

How were the informants selected?

- Convenience
- Random
- Volunteer
- Other

Does this study use a patient reported measure (patient reported outcome measure)?

- Yes
  - Survey
  - Focus group
  - Self-reported scale
  - Structured one-on-one interview
  - Internet
  - Other
    - Have this measure been previously validated?
      - Yes
      - No
      - Don’t know
      - No
      - Don’t know

Procedures for obtaining these voices:

- Training personnel involved
  - Who was trained? (text box)
- How was this training developed
  - Rules for the interaction
  - Pre-design interactions
  - Pre-design context/setting
  - Other

Challenges and questions for patients in research

- Ethics
- Capacity
- Power
- Trust/ Bias/ Representativeness
- Epistemology
- Terminology
- Interaction format
- Evaluation
- Reporting
- Ownership

How was the information used? (All these questions need a free text box to include the results)

- Agenda setting
- Funding
- Procedures
- Study design
- Recruitment
- Analysis
- Dissemination
- Others

Outcomes of incorporating patient voice (effect of the information):

- Author’s conclusions about the incorporation process and whether it led to a change in the conduct of research
- Actual data of the effect of the incorporation process

Barriers and Obstacles:

- Author conclusions
- Actual data

Congruence of patient opinion/voice

- With whom and how:

Involvement of the subject

- Definitions of the person:
  - Subject
  - Respondent
  - Participant
  - Stakeholder
  - Consultant
  - Partner
  - Researcher
- Person role:
  - Consent
  - Undergo
  - Review
  - Input
  - Dialogue
  - Collaborate
  - Generate

Recommendations:

-Authors recommendations /conclusions of How to incorporate patients’ voice into research? –--Actual

**Analysis**

- meta-narrative approach
- Analysis follows a framework that defines the key questions
- The included studies are evaluated until saturation for discrete themes and trends that can be mapped to outcomes.
- Differences in studies settings and characteristics can be used to explain differences in results (heterogeneity)

**
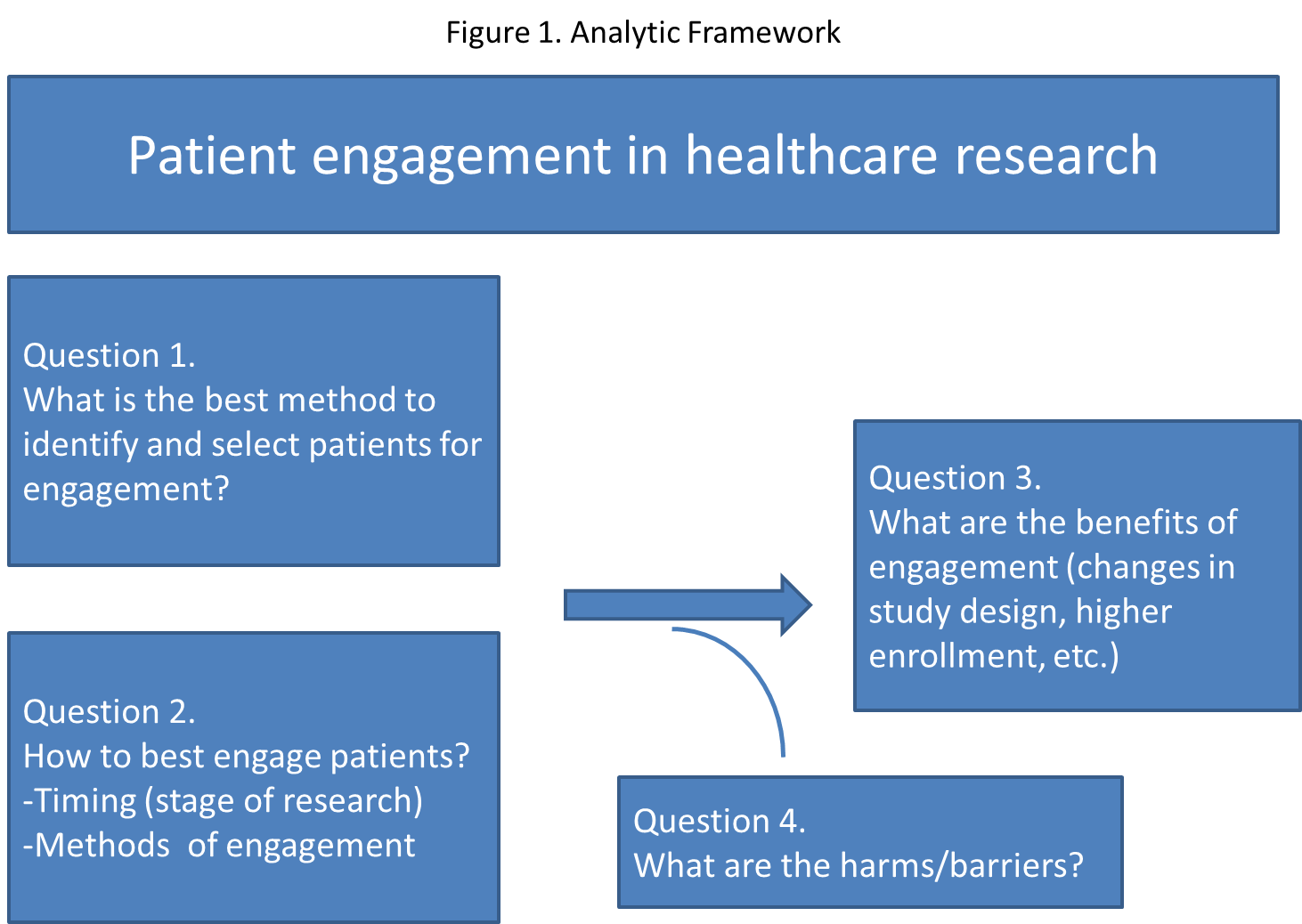
**

**Research phases proposed for patient engagement:**

1) Preparatory phase (agenda setting, prioritization of research topics and funding)

2) Execution phase (study design & procedures, study recruitment, data collection, and data analysis)

3) Translation phase (dissemination, implementation, and evaluation)

(2) **Search strategy**

**MEDLINE /EMBASE: From Inception to November 2011**

|  | |
| --- | --- |
| # | Search |
| 1 | patient participation/ or consumer participation/ or patient advocacy/ or consumer advocacy/ |
| 2 | patient centered care/ or ((patient* or consumer* or stakeholder* or user* or lay* or client* or citizen* or communit* or public or advoca* or carer* or caregiver* or surrogate* or parent* or relative) adj2 (important or perspective or centered or centred or participa* or collaborat* or partner* or voice* or unvoiced)).mp. [mp=protocol supplementary concept, rare disease supplementary concept, title, original title, abstract, name of substance word, subject heading word, unique identifier] |
| 3 | ((patient* or consumer* or stakeholder* or user* or lay* or client* or citizen* or communit* or public or advoca* or carer* or caregiver* or surrogate* or parent* or relative) adj2 (involv* or represent* or consult* or contribut* or engage* or activat* or opinion* or dialog* or partner* or input*)).mp. [mp=protocol supplementary concept, rare disease supplementary concept, title, original title, abstract, name of substance word, subject heading word, unique identifier] |
| 4 | *patient participation/ or *consumer participation/ or *patient advocacy/ or *consumer advocacy/ or (patient centered care or ((patient* or consumer* or stakeholder* or user* or lay* or client* or citizen* or communit* or public or advoca* or carer* or caregiver* or surrogate* or parent* or relative) adj2 (important or perspective or centered or centred or participa* or collaborat* or partner* or voice* or unvoiced))).ti,ab. or ((patient* or consumer* or stakeholder* or user* or lay* or client* or citizen* or communit* or public or advoca* or carer* or caregiver* or surrogate* or parent* or relative) adj2 (involv* or represent* or consult* or contribut* or engage* or activat* or opinion* or dialog* or partner* or input*)).ti,ab. or patient-centered care/ |
| 5 | exp evaluation studies as topic/ or exp methods/ |
| 6 | 4 and 5 |
| 7 | 6 and (outcome*.mp. or quality of life/ or patient preferences/ or risk assessment/ or patient satisfaction/) [mp=protocol supplementary concept, rare disease supplementary concept, title, original title, abstract, name of substance word, subject heading word, unique identifier] |
| 8 | 6 and ((utilities or values or empiric* or feedback* or communication*).mp. or health priorities/ or research priorities/) [mp=protocol supplementary concept, rare disease supplementary concept, title, original title, abstract, name of substance word, subject heading word, unique identifier] |
| 9 | 6 and exp clinical trials as topic/ |
| 10 | 6 and (physician-patient relations/ or patient acceptance of health care/) |
| 11 | Health Services Research/ or Needs Assessment/ |
| 12 | 6 and 11 |
| 13 | 6 and agenda*.mp. [mp=protocol supplementary concept, rare disease supplementary concept, title, original title, abstract, name of substance word, subject heading word, unique identifier] |
| 14 | 4 and *research design/ and (choice behavior/ or cooperative behavior/) |
| 15 | 6 and (choice behavior/ or cooperative behavior/) |
| 16 | 7 or 8 or 10 or 12 or 13 or 15 |
| 17 | 16 and ((panel* or jury or juries or forum).mp. or qualitative research/ or interview*.mp.) [mp=protocol supplementary concept, rare disease supplementary concept, title, original title, abstract, name of substance word, subject heading word, unique identifier] |
| 18 | 16 and (recruit* or participat* or "focus group*" or instrument* or scale* or questionnaire* or consultant* or questionnaire* or survey* or interview* or "nominal group" or delphi*).mp. [mp=protocol supplementary concept, rare disease supplementary concept, title, original title, abstract, name of substance word, subject heading word, unique identifier] |
| 19 | 17 or 18 |
| 20 | 16 and observation*.mp. [mp=protocol supplementary concept, rare disease supplementary concept, title, original title, abstract, name of substance word, subject heading word, unique identifier] |
| 21 | (19 or 20) and exp clinical trial as topic/ |
| 22 | 19 or 20 |
| 23 | limit 22 to (consensus development conference or consensus development conference, nih or multicenter study or "research support, american recovery and reinvestment act" or research support, nih, extramural or research support, nih, intramural or research support, non us gov't or research support, us gov't, non phs or research support, us gov't, phs) |
| 24 | 22 and (*patient satisfaction/ or *consumer satisfaction/ or *patient-center care/ or *patient preferences/) |
| 25 | 22 and (technology assessment, biomedical/ or community-based participatory research/ or px.fs.) |
| 26 | 21 or 24 or 25 |
| 27 | 23 and 2 |
| 28 | 26 or 27 |
| 29 | limit 28 to (comment or editorial or interview or introductory journal article or legislation or letter or news or newspaper article or patient education handout or retracted publication or "retraction of publication") |
| 30 | 28 not 29 |
| 31 | limit 30 to humans |
| 32 | 31 not animals/ |
| 33 | ((patient* or consumer* or stakeholder* or user* or lay* or client* or citizen* or communit* or public or advoca* or carer* or caregiver* or surrogate* or parent* or relative) adj2 (important or perspective or centered or centred or participa* or collaborat* or partner* or voice* or unvoiced)).ti,ab. |
| 34 | ((patient* or consumer* or stakeholder* or user* or lay* or client* or citizen* or communit* or public or advoca* or carer* or caregiver* or surrogate* or parent* or relative) adj2 (involv* or represent* or consult* or contribut* or engage* or activat* or opinion* or dialog* or partner* or input*)).ti,ab. |
| 35 | (24 or 33 or 34) and 32 |

| EBM Reviews: Cochrane Methodology Register 4th Quarter 2011 | | | |
| --- | --- | --- | --- |
| # | Search | Results | Search Type |
| 1 | (participat* adj2 research).mp. [mp=title, abstract, subject heading word] | 141 | Advanced |
| 2 | "CMR: Evaluation methodology - patient involvement".kw. | 521 | Advanced |
| 3 | "CMR: Other methodology - patient based outcome measures".kw. | 252 | Advanced |
| 4 | (design* or planning or priorit* or agenda* or participat*or decision*).mp. and (2 or 3) [mp=title, abstract, subject heading word] | 244 | Advanced |
| 5 | 2 or 3) and (perspective* or preference*).mp. [mp=title, abstract, subject heading word] | 128 | Advanced |
| 6 | 5 not 4 | 1087 | - |

**PsycINFO: 1967 to November Week 3 2011**

| # | Search | Results | Search Type |
| --- | --- | --- | --- |
| 1 | client participation | 1087 | Advanced |
| 2 | experimentation/ or exp consumer research/ or exp interdisciplinary research/ or exp qualitative research/ or exp experimental design/ or exp methodology | 147651 | Advanced |
| 3 | 1 and 2 | 128 | Advanced |
| 4 | 1 and (priorit* or participatory or engage* or planning or design* or perspective* or preference*).mp. [mp=title, abstract, heading word, table of contents, key concepts, original title, tests & measures] | 594 | Advanced |
| 5 | 3 or 4 655 | 655 | Advanced |
| 6 | limit 5 to (all journals and human) | 545 | Advanced |
| 7 | 1 and (agenda* or involve*).mp. [mp=title, abstract, heading word, table of contents, key concepts, original title, tests & measures] | 413 | Advanced |
| 8 | limit 7 to (all journals and human) | 336 | Advanced |
| 9 | 6 or 8 646 | 646 | Advanced |
| 10 | 9 and outcome*.mp. [mp=title, abstract, heading word, table of contents, key concepts, original title, tests & measures] | 155 | Advanced |
| 11 | 9 and (2 or methodol*.mp.) [mp=title, abstract, heading word, table of contents, key concepts, original title, tests & measures] | 124 | Advanced |
| 12 | 10 or 11 | 252 | Advanced |
| 13 | *client participation/ and 12 | 202 | - |

**CINAHL From Inception to November 2011**

| # | Search | Results | Search Mode | Search screen |
| --- | --- | --- | --- | --- |
| 1 | input* OR perspective* OR involve* OR planning OR design* OR outcome* | 529181 | Boolean/Phrase | - |
| 2 | (MM "consumer participation" OR "consumer advocacy") | 6819 | Boolean/Phrase | Advanced |
| 3 | (MH "Patient Centered Care") | 8236 | Boolean/Phrase | Advanced |
| 4 | (MH "Action Research") | 2501 | Boolean/Phrase | Advanced |
| 5 | S2 and S4 | 91 | Boolean/Phrase | Advanced |
| 6 | (MH "Research+") OR (MH "Behavioral Research") OR (MH "Medical Practice, Research-Based") | 803685 | Boolean/Phrase | Advanced |
| 7 | (MH "Study Design+") | 407274 | Boolean/Phrase | Advanced |
| 8 | S2 AND (S6 OR S7) | 2571 | Boolean/Phrase | Advanced |
| 9 | method* | 502921 | Boolean/Phrase | Advanced |
| 10 | S8 and S9 | 1120 | Boolean/Phrase | Advanced |
| 11 | S8 and S9 Limiters - Exclude MEDLINE records | 224 | Boolean/Phrase | Advanced |

**PubMed**

| # | Connector | Search |
| --- | --- | --- |
| 1 | **-** | (patient participation/methods[majr] |
| 2 | OR | consumer participation/methods[majr]) |
| 3 | AND | (clinical trials as topic[mesh]) |
| 4 | OR | (practice guidelines as topic[mesh]) |
| 5 | OR | (health services research[mesh]) |
