# Supplementary material for: Patient engagement in research: a systematic review
Source: BMC Health Serv Res. 2014 Feb 26;14:89. doi: 10.1186/1472-6963-14-89 (PMC3938901; doi:10.1186/1472-6963-14-89)
Supplement: Additional file 3: Table S1 — Systematic reviews. Table S2. Original studies. Table S3. Quality assessment of the original studies. Table S4. Initiatives and patient organizations identified by the environmental scan. [file 1472-6963-14-89-S3.docx]

**Additional file 3**

**Table S1: Systematic reviews:**

| ***Study Name (Author name, year)*** | ***Aim*** | ***Summarized literature*** | ***Main findings*** |
| --- | --- | --- | --- |
| ***Boote, 2010[***[***1***](#_ENREF_1)***]*** | To review published case examples of public involvement in primary research design. | 7 studies in various health topics | Group meetings are the most common method of public involvement. Contributions that members of the public made to research design may include review of consent procedures and patient information sheets, outcome suggestions, review of acceptability of data collection procedures, and recommendations on the timing of potential participants into the study and the timing of follow-up. |
| ***Brett 2010[***[***2***](#_ENREF_2)***]*** | To examine the conceptualization, measurement, impact and outcomes of patient and public involvement in health and social care research. In addition, economic evaluations were also sought in order to understand the financial impact of patient and public involvement activity. | 98 studies in various health topics | There is an emerging and important evidence of the impact of patient engagement on health care research but with relatively little conceptualization and theoretical development in the field. It also described poor quality of reporting as a one of the most important barriers restricting our understanding of the impact of patient engagement in research. |
| ***Hussain-Gambles, 2004[***[***3***](#_ENREF_3)***]*** | To Investigate how South Asian patients conceptualize the notion of clinical trials | 6 clinical trials in cancer and surgery. The review was supplemented by three qualitative interview studies | Factors that affect patient participation of clinical trial include health benefits, effective follow-up, clinician influence, communication style of doctor/nurse, and satisfaction with previous experience.  There are more similarities than differences in attitudes towards clinical trial participation between the South Asian and the general population.  Important decisions, such as participation in clinical trials, are likely to be made by those family members who are fluent in English and younger. |
| ***Legare, 2011[***[***4***](#_ENREF_4)***]*** | to identify key components of patient and public involvement programs used in developing and implementing clinical practice guidelines. | 71 studies in various health topics | The most listed reason for patient engagement was to incorporate patients’ values or perspectives. Methods used to recruit participants included soliciting through patient/public organizations, sending invitations, and receiving referrals and recruits from clinicians. Engagement methods included taking part in a working group, workshop, meeting, seminar, literature review, or consultation. |
| ***Mockford, 2012[***[***5***](#_ENREF_5)***]*** | To identify the impact of patient and public involvement on UK National Health Service healthcare services and to identify the economic cost | 42 studies in various health topics | There is little evidence of any economic analysis of the costs involved, poor quality of reporting, little theoretical or conceptual underpinning, lack of measurement and evaluation; and overall weak evidence base to support patient and public engagement. |
| ***Nilsen, 2006[***[***6***](#_ENREF_6)***]*** | To assesses the effects of consumer involvement and compare different methods of involvement in developing healthcare policy and research, clinical practice guidelines, and patient information material. | 6 randomized controlled trials in various health topics | There is insufficient data to evaluate the impact of patient engagement. The engagement is feasible in most of the included randomized controlled trials. |
| ***Oliver, 2004[***[***7***](#_ENREF_7)***]*** | To look at the process and outcomes of identification and prioritization and to identify the barriers and facilitators to meaningful participation by consumers | 286 documents in various health topics | Methods of engagement depend on the question being asked, tasks needed and consumer characteristics. More success might be expected if research programs embarking on collaborations approach well-networked consumers and provide them with information, resources and support to empower them in key roles. Consultations should engage consumer groups directly and repeatedly in facilitated debate; when discussing health services research, more resources and time are required if consumers are drawn from groups whose main focus of interest is not health. |
| ***Stewart,2011[***[***8***](#_ENREF_8)***]*** | To identify important areas of research, question for research and tools for assessment using patients' and clinicians' input | 250 studies in various health topics | The literature describes different methods for patients and clinicians to contribute to research agendas. Patients and clinicians may identify research questions together by engaging participants directly and repeatedly in facilitated debates and formal decision-making procedures. |

**Table S2: Original studies:**

| ***Study Name (Author’s name, year)*** | ***Design*** | ***Goal*** | ***Engagement method*** | ***Study settings*** | ***Engaged person(s)*** | ***Engaged person(s)' role*** | ***Sample selection*** |
| --- | --- | --- | --- | --- | --- | --- | --- |
| ***Randomized controlled trials*** | | | | | | | |
| ***Atkinson, 2011[***[***9***](#_ENREF_9)***]*** | Randomized controlled trial | To investigate the effects of changes in an informatics application on patients' participation | Survey, Structured one on one interview | Breast cancer | Patients | Subject  Consent, Undergo | Convenience |
| ***Daugherty, 1995[***[***10***](#_ENREF_10)***]*** | Randomized controlled trial | To describe a survey of cancer patients and their physicians to understand some of the complex issues related to the participation of cancer patients in phase I trials | Structured one on one interview | Cancer | Patients | Participant  Input | Convenience |
| ***Edwards, 2011[***[***11***](#_ENREF_11)***]*** | Randomized controlled trial | To demonstrate how consulting parents about the design of a study led to the design and successful delivery of a Randomized controlled trial of osteopathy for children with cerebral palsy | Structured one on one interview | Cerebral palsy | Surrogate | Participant  Input | Convenience |
| ***Koops, 2002[***[***12***](#_ENREF_12)***]*** | Randomized controlled trial | To determine whether consumer involvement would help to solve some of the ethical problems associated with research into thrombolysis for acute ischemic stroke, with its inherent risk of fatal intracranial hemorrhage | Survey, Focus group | Acute ischemic stroke | Patients | Respondent , Participant  Review, Input, Dialogue | Convenience |
| ***Marsden, 2004[***[***13***](#_ENREF_13)***]*** | Randomized controlled trial | To show breast cancer patient involvement in the design of a study | Focus group | Breast cancer | Patients, stakeholder and health professionals | Consultant  Input | Convenience |
| ***Shagi, 2008[***[***14***](#_ENREF_14)***]*** | Randomized controlled trial | To investigate the feasibility of a participatory model of community liaison among an occupational cohort of women at high-risk of HIV and sexually-transmitted infections in Mwanza City, northwest Tanzania in preparation for a Phase III vaginal microbicide trial | Meetings with community members | HIV high risk population | Patients | Stakeholder  Input | NR |
| ***Swartz, 2004[***[***15***](#_ENREF_15)***]*** | Randomized controlled trial | To describe the implementation and baseline data of an inner-city community based participatory research clinical trial designed to test the effectiveness of a pollutant and allergen control strategy on children's asthma morbidity | NA | Asthma | Surrogate, community organization members, school principals, pastor, nun, health professionals | Stakeholder  Input | Convenience |
| ***Observational studies*** | | | | | | | |
| ***Andejeski, 2002[***[***16***](#_ENREF_16)***]*** | Single cohort | To evaluate the impact of having breast cancer survivors with advocacy experience (consumers) participate as voting members of scientific review panels for proposals on breast cancer research. | Survey | Breast cancer | Patients | Participant  Review | Convenience |
| ***Bigrigg, 1999[***[***17***](#_ENREF_17)***]*** | Cross sectional study | To assess patient's views of commercial clinical trials | Survey | Contraceptive methods | Patients | Subject  Consent | Convenience |
| ***Caron-Flinterman, 2005b[***[***18***](#_ENREF_18)***]*** | Cross sectional study | To assess the ability of patients to prioritize research in a well-argued way. | Survey, Focus group, feedback meeting | Asthma and COPD patients | Patients | Respondent  Input, Dialogue | Random |
| ***Cashman, 2008[***[***19***](#_ENREF_19)***]*** | Case report | Describes 4 cases where community members participated in data analysis, interpretation, or both, | Focus group, advisory meetings | Community involvement | Community members, health professionals | Partner  Dialogue | NA |
| ***Crowe, 2008[***[***20***](#_ENREF_20)***]*** | Case report | To demonstrate concrete examples of ways in which community members can be involved in all stages of research | Survey, Community meetings | Occupational and environmental health in the Hispanic agricultural community | Patients, Community organizations | Consultant  Collaborate | Volunteer |
| ***Davison, 2008[***[***21***](#_ENREF_21)***]*** | Cross sectional study | To identify factors that patients with prostate cancer believe to be important determinants in their decisions about future enrolment in clinical trials. | Survey | Cancer | Patients | Subject, Participant  Consent, Undergo | Convenience |
| ***Dencker, 1986[***[***22***](#_ENREF_22)***]*** | Cross sectional study | To determine if research related values and priorities differed between patients and controls | Survey | Mental health | Patients | Participant  Review | Convenience |
| ***Doyle, 2010[***[***23***](#_ENREF_23)***]*** | Case report | To learn from a CBPR project. | Survey, Focus group | Elderly people | Patients | Respondent  Undergo | Convenience, Volunteer |
| ***Freysteinson, 2010[***[***24***](#_ENREF_24)***]*** | Case report | To describe the use of a community consultation ethical framework in the pre-research stage of investigating the experience of viewing oneself in a mirror after mastectomy | Structured one on one interview, telephone and e mail communication, group forums | Breast cancer | Patients, nurses and health care providers | Respondent  Input | NR |
| ***Hanley, 2001[***[***25***](#_ENREF_25)***]*** | Single cohort | To assess the extent to which consumers are involved in the work of clinical trial in the United Kingdom | Survey | Health research in general | Clinical trial coordinating centers | Participant  Input | NR |
| ***Jenkins, 2002[***[***26***](#_ENREF_26)***]*** | Cross sectional study | To examine whether there is a preferred way to describe the randomization process that may facilitate discussions about clinical trials of cancer therapy. | Survey | Cancer | Patients | Subject, Researcher  Review, Input | Convenience |
| ***Johnston, 2008a[***[***27***](#_ENREF_27)***]*** | Case report | To discuss engaging patients in cancer and palliative care research | Workshop | Cancer | Patients | Subject, Participant, Partner  Consent, Review, Input, Collaborate | NA |
| ***Jones, 2006[***[***28***](#_ENREF_28)***]*** | Cross sectional study | To assess cancer patients' knowledge and attitudes towards clinical trials. | Survey | cancer | Patients | Respondent  Input | Random |
| ***Kamps, 1987[***[***29***](#_ENREF_29)***]*** | Single cohort | To discuss parental attitude and perceptions of a child's responsibilities on experimental therapy | Survey | pediatric cancer | Surrogate | Respondent  Undergo | Convenience |
| ***Kelson 1999[***[***30***](#_ENREF_30)***]*** | Cross sectional study | To identify the extent to which the Cochrane Collaboration involves consumers as members of Cochrane Review Groups | Survey | NA | Cochrane Review Groups | NR | NR |
| ***Langston, 2005[***[***31***](#_ENREF_31)***]*** | Case report | To outline the experiences of an integrated relationship between the organizers of a clinical trial and a consumer organization. | NA | Paget Disease | Patients | Consultant  Input | Volunteer |
| ***Leinisch-Dahlke, 2004[***[***32***](#_ENREF_32)***]*** | Single cohort | To discuss patient preference compared with the expert preference regarding clinic trials and drug therapy | Survey | Headache | Patients | Respondent  Input | Convenience |
| ***Madsen, 2000[***[***33***](#_ENREF_33)***]*** | Single cohort | To investigate the preferred extent of written information in clinical trials among potential and actual trial participants | Survey | Patients in an outpatient clinic and clinical trial participants | Patients | Subject  Undergo | Random and convenience sample |
| ***Meropol, 2003[***[***34***](#_ENREF_34)***]*** | Cross sectional study | To describe and compares the perceptions of cancer patients and their physicians regarding phase I clinical trials. | Survey | Cancer patients | Patients, | Respondent  Undergo | Convenience |
| ***Minogue, 2010[***[***35***](#_ENREF_35)***]*** | Case report | Describe current state of patient involvement in research in UK | NA | All research in UK | Patients, Researcher | Consultant, Researcher  Collaborate | NA |
| ***Noe, 2007[***[***36***](#_ENREF_36)***]*** | Single cohort | To evaluate whether community-based participatory research principles might influence an individual’s decision to participate in research | Survey, Focus group | American Indian | Patients | Respondent  Undergo, Dialogue | Convenience |
| ***Sood, 2009[***[***37***](#_ENREF_37)***]*** | Single cohort | To assess attitudes of patients about participation in clinical trials | Survey | Health research in general | Patients | Respondent  Undergo | Convenience |
| ***Thomas, 1999[***[***38***](#_ENREF_38)***]*** | Cross sectional study | To examine the reasons for women's participation in breast screening | Survey | Breast cancer | Patients | Respondent  Undergo | Convenience |
| ***White, 2005[***[***39***](#_ENREF_39)***]*** | Single cohort | To increase the understanding of decision making about cancer management by men with prostate cancer who have decided to forgo conventional treatment | Survey | Prostate cancer | Patients | Stakeholder  Dialogue | Convenience |
| ***Qualitative studies*** | | | | | | | |
| ***Abma, 2005[***[***40***](#_ENREF_40)***]*** |  | To describes a responsive-constructivist approach to evaluate the aims and features of patient participation and learning experience. | Structured one on one interview, online patient forum, workshops | Spinal cord injury | Patients | Partner Collaborate | Convenience |
| ***Abma, 2010[***[***41***](#_ENREF_41)***]*** |  | To develop a methodology for health research agenda setting processes grounded in the notion of participation as dialogue. | Structured one on one interview | Spinal cord injury, neuromuscular diseases, renal failure, asthma ⁄chronic  Obstructive pulmonary disease, burns, diabetes and intellectual disabilities. | Members of patient organizations | NA | NA |
| ***Ågård, 2001[***[***42***](#_ENREF_42)***]*** |  | To investigate how patients included in trials on treatment in the early phase of acute myocardial infarction experience the consent procedure | Semi structured interviews | Myocardial infarction | Patients | Participant  Input, Dialogue | Convenience |
| ***Ard, 2005[***[***43***](#_ENREF_43)***]*** |  | To identify unique variables for African Americans that might limit the effectiveness of behavioral interventions in clinical trials | Focus group | African Americans | Patients | Participant  Dialogue | NR |
| ***Asai, 2004[***[***44***](#_ENREF_44)***]*** |  | To explores laypersons’ attitudes towards and experiences of medical research | Focus group | Health research in general | Patients, physicians | Respondent  Undergo | Convenience |
| ***Australian Government[***[***45***](#_ENREF_45)***]*** |  | To present a Model Framework for Consumer and community Participation in Health and Medical Research | Participatory methods | Participation in Health and Medical Research | community members | Partner  Generate | NR |
| ***Brody, 2009[***[***46***](#_ENREF_46)***]*** |  | To examine the impact of physician-investigator relationships and clinical research-participation recommendations on family decisions to enroll adolescents in a asthma RCT | Self-reported scale | Adolescent asthma | Patients, Relative | Stakeholder  Dialogue | Volunteer |
| ***Campbell, 1998[***[***47***](#_ENREF_47)***]*** |  | To learn collaboratively about health care service provision from the standpoint of people with disabilities | NR | Care of patients with disabilities | Patients, health providers, research staff and university faculty | Researcher  Generate | NR |
| ***Carey, 1992[***[***48***](#_ENREF_48)***]*** |  | To describe the author's use of qualitative data in the refinement of research in a medical setting | Focus group | HIV infected patients | Patients | Participant  Collaborate | NR |
| ***Carey, 2001[***[***49***](#_ENREF_49)***]*** |  | To describe the experience of 45 outpatients who recently completed their participation in a randomized clinical trial | Structured one on one interview | Severe and persistent mental illness | Patients | Participant  Input | Convenience |
| ***Caron-Flinterman, 2005a[***[***50***](#_ENREF_50)***]*** |  | To discuss the validity of patients’ experiential knowledge in the context of biomedical research processes. | Structured one on one interview | Health research in general | Patients, scientists and patients’ organizations | Participant, Researcher  Input | Convenience |
| ***Carr, 2003[***[***51***](#_ENREF_51)***]*** |  | To explore the patient’s perspective of outcomes in rheumatoid arthritis to identify which outcomes are important to patients | Focus group | Rheumatoid arthritis | Patients | Respondent  Dialogue | Convenience |
| ***Casarett, 2001[***[***52***](#_ENREF_52)***]*** |  | To define the endpoints of pain research that are important to patients with chronic pain and to identify clinical and demographic variables that are associated with patients’ choices of endpoints. | Structured one on one interview | Pain | Patients | Participant  Dialogue | Convenience |
| ***Chenoweth, 1998[***[***53***](#_ENREF_53)***]*** |  | To describe the way in which family careers participated with day-care staff to design a therapeutic program for people attending a dementia day care program. | NA | Dementia | Patients, Relative, day-care staffs | Subject, Researcher  Input, Dialogue | Convenience |
| ***Corneli, 2007[***[***54***](#_ENREF_54)***]*** |  | To learn the attitudes and concerns of the local community on participating in research | Focus group | HIV | Patients | Respondent  Undergo | Convenience |
| ***Cotterell, 2008[***[***55***](#_ENREF_55)***]*** |  | To evaluate the process and outcomes of service user involvement in the analysis of data | NA | Life limiting conditions, including cancer, COPD | Patients | Consultant  Input | Convenience |
| ***Cox, 1996[***[***56***](#_ENREF_56)***]*** |  | To describe the findings of a study that explored the psychosocial aspects of participation in early anticancer drug trials from the perspective of the patients. | Structured one on one interview | Cancer | Patients | Participant  Input | Convenience |
| ***Cox, 2000[***[***57***](#_ENREF_57)***]*** |  | To examine patient’s perceptions of participating in early phase anti-cancer drug trials | Survey, Structured one on one interview | Advanced cancer | Patients | Respondent , Participant  Input, Dialogue | Volunteer |
| ***Curry, 2006[***[***58***](#_ENREF_58)***]*** |  | To allow key staff and patients to be involved at all levels of the new nurse-led urgent care team (UCT). The project aimed to evaluate the impact of the UCT | Structured one on one interview | COPD patients | Patients | Respondent  Dialogue | Volunteer |
| ***Daly, 2009[***[***59***](#_ENREF_59)***]*** |  | Explore ways in which foster care children's values and preferences regarding research in which they would be involved. | Focus group | Foster care | Patients | Stakeholder  Dialogue | Convenience |
| ***Daugherty, 1999[***[***60***](#_ENREF_60)***]*** |  | To understand some of the complex issues related to the participation of cancer patients in phase I trials, and the perceptions of patients toward these trials | NA | Cancer | Patients | Subject  Consent | NA |
| ***Dellson, 2011[***[***61***](#_ENREF_61)***]*** |  | To describe patients' opinions about the written information used in 3 clinical trials for breast cancer | Survey Focus group | Breast cancer | Patients | Participant  Undergo, Input | Convenience |
| ***Dixon-Woods, 2006[***[***62***](#_ENREF_62)***]*** |  | To explore trial participants’ responses to receiving a summary of the results of a trial in pregnancy. | Structured one on one interview | ORACLE trial of antibiotics for preterm labor and preterm rupture of the membranes | Patients | Respondent  Dialogue | Volunteer |
| ***Eng, 2005[***[***63***](#_ENREF_63)***]*** |  | To examine the difference between the reasons for accepting and declining participation in a two-arm active treatment Randomized controlled trial comparing external beam radiation therapy vs. cryapy | Structured one on one interview | Prostate cancer | Patients | Participant  Input | Convenience |
| ***Featherstone, 1998[***[***64***](#_ENREF_64)***]*** |  | To explore trial participants' understandings of randomization. | Structured one on one interview | Benign prostatic disease. | Patients | Participant  Dialogue | Convenience |
| ***Fern, 2011[***[***65***](#_ENREF_65)***]*** |  | To involve young people in research. the report was focused on how the young people were involved to inform each stage of the action research | Participatory group methods | Involvement of young people in research | Young people | Participant  Dialogue | NR |
| ***Forbes, 2010[***[***66***](#_ENREF_66)***]*** |  | To assess women’s views of the design of randomized controlled trial | Focus group | Breast cancer | Patients | Respondent  Undergo | Convenience |
| ***Garber, 2007[***[***67***](#_ENREF_67)***]*** |  | To develop a questionnaire that measures attitudes and concerns about HIV treatment trials among HIV-infected African Americans. To determine actual participation rates and willingness to participate in future HIV treatment trials among HIV-infected African Americans | Survey | HIV-infected African-American adults | Patients | Respondent  Input | Volunteer |
| ***Gittelsohn, 2010[***[***68***](#_ENREF_68)***]*** |  | To develop a community-based chronic disease prevention program for Inuit in Nunavut, Canada | Structured one on one interview | Community involvement | Patients, community leaders and members, health and social service staff | Consultant  Collaborate | Convenience |
| ***Gooberman, 2008[***[***69***](#_ENREF_69)***]*** |  | To describe challenges and benefits of using citizen's juries as member of the public | Focus group | Primary health and social care | Citizens | Consultant  Dialogue | Convenience |
| ***Harper, 2000[***[***70***](#_ENREF_70)***]*** |  | To present a model to address the interactional process that occurs while developing a CBO. | CBO collaborative partnership model | AIDS/HIV | Patients | Partner, Researcher  Collaborate, Generate | NA |
| ***Higgins, D 2001[***[***71***](#_ENREF_71)***]*** |  | To describe the process of participatory research | NR | Urban health issues | Patients | Partner  Collaborate | NA |
| ***Hsu, 2010[***[***72***](#_ENREF_72)***]*** |  | To provide insight into the full range of meaningful outcomes experienced by patients who participate in clinical trials of complementary and alternative medicine therapies. | Structured one on one interview | Patients who participate in clinical trials | Patients | Respondent  Dialogue | Volunteer |
| ***Hutchison, 1998[***[***73***](#_ENREF_73)***]*** |  | To determine how cancer patients perceive phase I clinical trials in reference to trial participation and trial information received. | Structured one on one interview | Cancer | Patients | Participant  Input | Convenience |
| ***Irani, 2010[***[***74***](#_ENREF_74)***]*** |  | To describe the development of a research protocol on secondhand tobacco smoke exposure and chronic rhino sinusitis for a future population-based case control study using a participatory research model. | Focus group | Secondhand tobacco smoke exposure and chronic rhino sinusitis | Community members, health practioners, and researchers | Partner  Dialogue | Convenience |
| ***Jenkins, 2005[***[***75***](#_ENREF_75)***]*** |  | To identify the preferred and most disliked descriptions of randomization found in current cancer patients | Survey | Patients from cancer centers throughout the UK | Patients | Respondent  Input | Convenience |
| ***Jinks, 2009[***[***76***](#_ENREF_76)***]*** |  | To establish a community knee pain forum aimed at engaging stakeholders in design, dissemination and prioritization of knee pain research | Group meeting | Knee pain | Patients, health professional, community members, researchers | Stakeholder  Dialogue | Convenience |
| ***Karlawish, 2008[***[***77***](#_ENREF_77)***]*** |  | To examine the views of Alzheimer disease patients and their study partners on the ethics of proxy consent for clinical research | Structured one on one interview | Alzheimer's disease | Patients, Surrogate | Respondent  Dialogue | Volunteer |
| ***Karmaliani,2009[***[***78***](#_ENREF_78)***]*** |  | To share concerns about maternal depression, partner violence, and child functioning and the goal of offering an intervention (i.e., program) to the community to improve maternal mental health and child functioning | Focus group | Maternal depression and child health | Community leaders, agency directors | Partner  Collaborate | NA |
| ***Kelly, 2005[***[***79***](#_ENREF_79)***]*** |  | To describe background steps that researchers can use when conceptualizing and initiating a research project with community partners in participatory action research | NA | Health research in general | NA | NA | NR |
| ***King, 2009[***[***80***](#_ENREF_80)***]*** |  | describes the development of a 33-item, survey questionnaire measuring community members’ perceptions of the impact of research partnerships addressing health or social issues | Focus group | Health research in general | Researchers and community members | Consultant  Review, Dialogue | Convenience |
| ***Kirwan, 2003[***[***81***](#_ENREF_81)***]*** |  | To assess the outcomes of intervention in rheumatoid arthritis (RA) from the perspective of those who experience the disease themselves. | Focus group | Rheumatoid arthritis | Patients | Consultant  Generate | Convenience |
| ***Kirwan, 2005[***[***82***](#_ENREF_82)***]*** |  | To evaluate outcomes of intervention in rheumatoid arthritis (RA) from the perspective of those with RA | Focus group | Rheumatoid arthritis | Patients | Respondent  Undergo | Convenience |
| ***Lammers, 2004[***[***83***](#_ENREF_83)***]*** |  | To describe the establishment and function of a reference group established to guide the conduct of a research project examining the experiences of consumers and careers with psychiatric disability support services. | Focus group | Consumers and carers with psychiatric disability support services | Patients | Subject  Collaborate | NR |
| ***Lavender, 2009[***[***84***](#_ENREF_84)***]*** |  | To explore women's views of participation in a trial of planned cesarean birth vs. planned virginal birth. | Structured one on one interview | Delivery | Patients | Participant  Input | NA |
| ***Lindenmeyer, 2007[***[***85***](#_ENREF_85)***]*** |  | To determine what makes user involvement successful, effective and meaningful from the researcher standpoint | Semi-structured interviews | Diabetes | Researchers | Researcher  Generate | Extensive interaction with advisory group |
| ***Llewellyn-Thomas, 1989[***[***86***](#_ENREF_86)***]*** |  | To describe a method to test patients' attitudes towards the possible risks and benefits associated with any new treatment which has been incorporated into a clinical trial protocol. | Structured one on one interview | Breast cancer | Patients | Participant  Input | Convenience |
| ***MacKinnon, 2010[***[***87***](#_ENREF_87)***]*** |  | To describe the methodology and lessons learned from the collaboration with community-based organizations to explore how to measure the difficult-to-measure outcomes of participation in community-based programs | Structured one on one interview | Aboriginal | Patients | Participant  Input | Convenience |
| ***Manson, 2004[***[***88***](#_ENREF_88)***]*** |  | To illustrate successful strategies in working with American and Alaska Native communities in aging and health research | Secondary data analysis | Health research in general | NA | NA | Convenience |
| ***Martin, 2009[***[***89***](#_ENREF_89)***]*** |  | To determine the feasibility of engaging incarcerated women in CBPR and to identify by and with the women the health concerns that needed to be addressed. | Structured one on one interview and group interviews | Incarcerated women | Patients | Researcher  Generate | Volunteer |
| ***Maslin-Pro, 2003[***[***90***](#_ENREF_90)***]*** |  | To describe the experience of user involvement in health care research drawing on current UK health policy documents and an examination of the factors affecting recruitment to breast cancer clinical trials | Focus group | Breast cancer | Patients | Participant  Dialogue | NA |
| ***Mastwyk, 2002[***[***91***](#_ENREF_91)***]*** |  | To assess why the carers of people with Alzheimer’s disease seek the participation of their relatives in clinical trials | Survey | Alzheimer's disease | Relative | Subject  Consent | Volunteer |
| ***McQuiston, 2005[***[***92***](#_ENREF_92)***]*** |  | To generate preliminary data to be used to write a grant proposal | Focus group | HIV | Community leaders | Consultant  Collaborate | Convenience |
| ***Mease ,2007[***[***93***](#_ENREF_93)***]*** |  | To assess the core domains assessed in fibromyalgia studies | Focus group | Fibromyalgia | Patients | Respondent  Undergo | Convenience |
| ***Medd, 2005[***[***94***](#_ENREF_94)***]*** |  | To assess men’s experience of prostate biopsy | Survey, Semi-Structured interview shortly after their prostate biopsy | Men for needle biopsy of the prostate. | Patients | Respondent  Input, Dialogue | Volunteer |
| ***Milewa, 2008[***[***95***](#_ENREF_95)***]*** |  | To describe the issues of an advisory group established to provide lay perspectives on the work of the UK’s Medical Research Council | Structured one on one interview | Health research in general | Health professionals (researchers, clinicians, and members from health professional organizations ) | Respondent  Dialogue | Convenience |
| ***Mills, 2003[***[***96***](#_ENREF_96)***]*** |  | To explore patients’ perceptions of randomization and reasons for consent or refusal to participate in a clinical trial | Structured one on one interview | Prostate cancer | Patients | Participant  Input | Convenience |
| ***Minkler, 2002[***[***97***](#_ENREF_97)***]*** |  | To explore the use of participatory action research by and with a community of people with disabilities in addressing a polarizing issue in that community: death with dignity or physician-assisted suicide legislation. | Structured one on one interview | Patients with substantial physical Disabilities | Patients, community members | Participant  Input | NR |
| ***Moreno-Black, 2004[***[***98***](#_ENREF_98)***]*** |  | To describe the results of a study that was designed to examine some of the ways in which participants in a randomized double blind clinical trial perceived their participation in the clinical trial | Structured one on one interview | HIV | Patients | Participant  Input | Convenience |
| ***Morin, 2008[***[***99***](#_ENREF_99)***]*** |  | To understand the evolution of community advisory boards and community partnerships at international research sites conducting HIV prevention trials | Focus group | HIV prevention | Community advisory board members | Consultant  Collaborate | Volunteer |
| ***Murad, 2011[***[***100***](#_ENREF_100)***]*** |  | To examine patients’ preferences regarding the design of diabetes trials. | Survey | Diabetes | Patients | Respondent  Input | Random |
| ***Nair, 2004[***[***101***](#_ENREF_101)***]*** |  | To explore the consent preferences of patients whose health data are currently being used for research purposes. | Structured one on one interview | Health research in general | Patients | Participant  Input | Convenience |
| ***Ntshanga, 2010[***[***102***](#_ENREF_102)***]*** |  | To present a framework which describes the establishment and benefits of the community advisory board in the Inanda, Ntuzuma and KwaMashu area | Focus group | Tuberculosis | Patients | Stakeholder  Review, Generate | Convenience |
| ***Ong, 2003[***[***103***](#_ENREF_103)***]*** |  | To involve users in the design of a research project that aims at describing a 12-month course of low back pain in an adult population sample, and to determine how patient and professional perceptions of low back pain and its treatment relate to the use of health-care and to subsequent outcome | Focus group | Back pain | Patients | Consultant  Dialogue | Convenience |
| ***Paul, 2011[***[***104***](#_ENREF_104)***]*** |  | To identify the perceptions of professionals, patients and carers regarding prioritizing psychosocial research efforts about hematological cancers | Survey | Hematological cancers | Patients, carers and health professional | Input  Collaborate | NR |
| ***Plumb, 2008[***[***105***](#_ENREF_105)***]*** |  | To determine the relationship between the collaborative process of conducting the Community Research Collaboration projects and reported outcomes | Semi-structured interviews | Projects with underserved populations | Surrogate | Researcher  Collaborate, Generate | All significant members |
| ***Read, 2009[***[***106***](#_ENREF_106)***]*** |  | To address the factors that influence young adults decision to participate in health care research | Survey | Cancer | Patients, Surrogate | Respondent  Undergo | Random |
| ***Reddy, 2010[***[***107***](#_ENREF_107)***]*** |  | To examine within the South African HIV vaccine clinical trial environment: the purpose of the CABs; the structure and representivity of the CABs; the scope of power and authority of the CABs; and the level of independence of the CABs. | Focus group | HIV patients | Principal investigators, counselors, community liaison officers, recruiters and CAB members. | Participant, Stakeholder, Consultant  Input, Dialogue | Volunteer |
| ***Redwood, 2010[***[***108***](#_ENREF_108)***]*** |  | To evaluate the use of community-based participatory research principles led to more effective study design and implementation in a study in Alaska | Survey | Health disparities | Surrogate | Consultant, Partner  Collaborate | Convenience |
| ***Richards, 2002[***[***109***](#_ENREF_109)***]*** |  | To place community involvement at a high priority in the development of health intervention research through a NIH funding initiative in the Washington DC area | Focus group | Studies focused on infant mortality and low birth weight outcomes | Researcher | Researcher  Generate | NA |
| ***Roberts, 2000[***[***110***](#_ENREF_110)***]*** |  | To describe how individuals with serious mental illness and psychiatrists view ethically important aspects of biomedical research participation | Structured one on one interview | Schizophrenia | Patients, psychiatrists | Participant  Input | Convenience |
| ***Roberts, 2002[***[***111***](#_ENREF_111)***]*** |  | To examine the perspectives of schizophrenia patients and psychiatrists regarding medication washouts and placebo treatment conditions in clinical researches. | Structured one on one interview | Schizophrenia | Patients | Respondent  Input | Convenience |
| ***Roberts, 2004[***[***112***](#_ENREF_112)***]*** |  | To examine the perspectives and preferences regarding ethically important aspects of recruitment, consent, and debriefing of people with schizophrenia who volunteered for research protocols | Structured one on one interview | Schizophrenia | Patients | Participant  Input | Convenience |
| ***Roe, 1995[***[***113***](#_ENREF_113)***]*** |  | Document experience of grandmas as primary caregivers to grandchildren | Semi-structured interviews | Grandmothers serving as primary caregivers | Patients, Researchers and community advisory board | Partner  Dialogue | Convenience |
| ***Rogers, 1994[***[***114***](#_ENREF_114)***]*** |  | To determine the residential needs and preferences of two important groups: primary consumers of mental health services and family members of consumers. | Survey | Mental health | Patients and family members | Input  Dialogue | NR |
| ***Rosen, 2007[***[***115***](#_ENREF_115)***]*** |  | To explore whether overall satisfaction with clinical care was a function of participation in research. | Survey | Persons with mental illness | Patients | Respondent  Input | Convenience |
| ***Ross, 2005[***[***116***](#_ENREF_116)***]*** |  | To evaluate the perceptions of risk of falls of older people | Focus group | Risk of falls | Patients | Respondent  Undergo | Convenience |
| ***Saurbrey, 1984[***[***117***](#_ENREF_117)***]*** |  | To evaluate patient's attitudes towards medical trials | Structured one on one interview | Research involving humans | Patients | Subject  Consent | Random |
| ***Savage, 2006[***[***118***](#_ENREF_118)***]*** |  | to explore the culture of pregnancy and infant care among African American women | Focus group | Social topic | Patients, Nurses in the community | Partner  Dialogue, Collaborate | Convenience |
| ***Sayers, 2009[***[***119***](#_ENREF_119)***]*** |  | To understand patient groups’ perceptions and experiences of the Roche clinical trials registry | Survey | Oncology, osteoporosis, virology | Patients | Respondent  Review, Input, Dialogue | NA |
| ***Serrano-Aguilar, 2009[***[***120***](#_ENREF_120)***]*** |  | To describe a study of incorporating patients in developing the early stages of a systematic review process | Delphi methods | Degenerative ataxias | Patients | Participant  Dialogue | Convenience |
| ***Shilling, 2011[***[***121***](#_ENREF_121)***]*** |  | to optimize recruitment of children to clinical trials | Survey, Structured one on one interview | Clinical trials in general | Relative, practitioners | Respondent  Undergo | Convenience |
| ***Slomka, 2008[***[***122***](#_ENREF_122)***]*** |  | To explore motivations of underserved African American drug users to participate in research | Structured one on one interview | HIV/AIDS | Patients | Subject, Participant  Consent | Convenience |
| ***Smith, 2007[***[***123***](#_ENREF_123)***]*** |  | To examine African American women's thoughts and perceptions about the clinical research process and about participation in the University of Michigan Women's Health Registry research database | Focus group | African American women | Patients | Participant  Dialogue | Convenience |
| ***Snowdon, 2006[***[***124***](#_ENREF_124)***]*** |  | To explore the pace of decision-making for 78 parents associated with clinical trials in the UK | Structured one on one interview | Recruitment of children in trials | Surrogate | Participant  Input | Convenience |
| ***Snowdon, 1997[***[***125***](#_ENREF_125)***]*** |  | To describe the views of parents who consented that their critically ill newborn baby should be enrolled in a neonatal trial |  | Randomized controlled trials involving neonates | Parents | Respondent  Input, dialogue | NR |
| ***Staniszewska, 2007[***[***126***](#_ENREF_126)***]*** |  | To involve users in the development of a research bid to examine parents’ experiences of having a pre-term baby | Meetings with patients' parents and researchers | Pre-term child | Surrogate | Partner  Collaborate | Convenience |
| ***Staniszewska, 2011[***[***127***](#_ENREF_127)***]*** |  | to develop the Guidance for Reporting Involvement of Patients and Public checklist to enhance the quality of patient and public involvement reporting | Thematic analysis | Patient and public involvement in research | NA | NA | NA |
| ***Stewart, 2006[***[***128***](#_ENREF_128)***]*** |  | To raise the importance of Indigenous participation in the ethical review process | Survey | Research involving humans | Indigenous human research ethics committees | Partner  Review | All the committees |
| ***Stewart, 2009[***[***129***](#_ENREF_129)***]*** |  | To discuss the participatory development and implementation of a community research workshop, the community and organizational contexts, the content of the workshop, and lessons learned. | Focus group | Health research in general | Community members | Partner  Collaborate | Convenience |
| ***Stirman, 2010[***[***130***](#_ENREF_130)***]*** |  | To describe the collaborative process, key challenges, and strategies employed to meet the goals of the first phase of the IP-RISP grant. | Focus group | Depression | Patients | Stakeholder, Partner, Researcher  Review, Dialogue | NA |
| ***Sugarman,1998[***[***131***](#_ENREF_131)***]*** |  | to determine patients' attitudes about medical research | Focus group | Cancer and heart disease | Patients | Respondent  Consent, Undergo | Convenience |
| ***Sullivan, 2005[***[***132***](#_ENREF_132)***]*** |  | To describe specific ways in which a participatory action research operationalized and how community participation shaped various stages of the research | Meetings with Project Advisory Group | Domestic violence | Patients, members from community organizations, community advise | Partner  Dialogue | Convenience |
| ***Thurston, 2005[***[***133***](#_ENREF_133)***]*** |  | To describe the theoretical framework developed to evaluate public participation in the context of regionalized health governance. | Survey, Structured one on one interview, case study | Public involvement in health care | Patients | Consultant  Collaborate | NR |
| ***Timotijevic, 2007[***[***134***](#_ENREF_134)***]*** |  | The objectives of the current research are to evaluate two deliberative methods—citizens’ jury and citizens’ workshop for both their process and outcome | Survey | Health research in general | Patients | Participant  Input, Dialogue | NR |
| ***Tischler, 2010[***[***135***](#_ENREF_135)***]*** |  | To explore the experience of both professionals and patients taking part in a research project that strove to be collaborative and patient-centered | Survey, Focus group | patient centered outcome | Patients | Respondent  Undergo | Convenience |
| ***Tobin, 2002[***[***136***](#_ENREF_136)***]*** |  | To evaluate the level, extent and quality of consumer participation, and to examine differences between services with different resource commitments | Structured one on one interview | Mental health | Patients | Participant  Input | Volunteer |
| ***Van Olphen, 2009[***[***137***](#_ENREF_137)***]*** |  | To evaluate the participatory approach in translating scientific findings from two key projects to the public. | Focus group | Breast cancer | Researchers, community members, and the hosting organization | Researcher  Generate | NR |
| ***van Staa, 2010[***[***138***](#_ENREF_138)***]*** |  | To evaluate feasibility, benefits and limitations of a participatory research project involving chronically ill adolescents as co-researchers | Structured one on one interview | Chronically ill adolescents | Patients | Participant, Researcher  Input, Generate | Convenience |
| ***Wersch, 2001[***[***139***](#_ENREF_139)***]*** |  | To evaluate consumer’s involvement in clinical guidelines development. | NA | Health research in general | Patients | Stakeholder  Dialogue | NR |
| ***White, 2008[***[***140***](#_ENREF_140)***]*** |  | To determine if patients with advanced cancer are interested in participation in palliative care research and the importance of demographic factors in decision making | Survey | Patients with an advanced disease and limited prognosis is | Patients, Relative | Respondent  Dialogue | Random |
| ***Wright, 1997[***[***141***](#_ENREF_141)***]*** |  | To determine the reliability, validity, and responsiveness of the Patient-Specific Index and to compare different methods of combining patients' ratings of the severity and importance of their complaints | Survey | Patients with total hip arthroplasty | Patients | Respondent  Input | Convenience |
| ***Zullino, 2003[***[***142***](#_ENREF_142)***]*** |  | To evaluate the general readiness of psychiatric inpatients to give their consent to different forms of studies and to assess their reasons for accepting or refusing a hypothetical participation. | Structured one on one interview | Psychiatric diseases | Patients | Consultant  Dialogue | Convenience |

*NA: Not applicable, NR: Not reported

**Table S3: Quality assessment of the original studies:**

| ***Study name*** | ***Aims were clearly stated*** | ***Research design was appropriate*** | ***Recruitment strategy was appropriate*** | ***Data collection method was appropriate*** | ***Ethical issues were addressed*** | ***Data analysis sufficiently rigorous*** | ***Findings were clearly stated*** |
| --- | --- | --- | --- | --- | --- | --- | --- |
| ***Randomized controlled trials*** | | | | | | | |
| ***Atkinson, 2011[***[***9***](#_ENREF_9)***]*** | Yes | Yes | Yes | Yes | Yes | Yes | Yes |
| ***Daugherty, 1995[***[***10***](#_ENREF_10)***]*** | Yes | Yes | Yes | Yes | Yes | Yes | Yes |
| ***Edwards, 2011[***[***11***](#_ENREF_11)***]*** | Yes | Yes | Yes | Yes | Yes | Yes | Yes |
| ***Koops, 2002[***[***12***](#_ENREF_12)***]*** | Yes | Yes | Yes | Yes | Yes | Yes | Yes |
| ***Marsden, 2004[***[***13***](#_ENREF_13)***]*** | Yes | Yes | Yes | Yes | Yes | Yes | Yes |
| ***Shagi, 2008[***[***14***](#_ENREF_14)***]*** | Yes | Yes | Yes | NR/Unclear | Yes | NR/Unclear | Yes |
| ***Swartz, 2004[***[***15***](#_ENREF_15)***]*** | Yes | Yes | Yes | Yes | Yes | NR/Unclear | Yes |
| ***Observational studies*** | | | | | | | |
| ***Andejeski, 2002[***[***16***](#_ENREF_16)***]*** | Yes | Yes | NR/Unclear | Yes | Yes | NR/Unclear | Yes |
| ***Bigrigg, 1999[***[***17***](#_ENREF_17)***]*** | Yes | Yes | Yes | Yes | Yes | NR/Unclear | Yes |
| ***Caron-Flinterman, 2005b[***[***18***](#_ENREF_18)***]*** | Yes | Yes | Yes | Yes | Yes | Yes | Yes |
| ***Cashman, 2008[***[***19***](#_ENREF_19)***]*** | Yes | Yes | NR/Unclear | Yes | Yes | NR/Unclear | Yes |
| ***Crowe, 2008[***[***20***](#_ENREF_20)***]*** | Yes | Yes | Yes | Yes | Yes | NR/Unclear | Yes |
| ***Davison, 2008[***[***21***](#_ENREF_21)***]*** | Yes | Yes | Yes | Yes | Yes | Yes | Yes |
| ***Dencker, 1986[***[***22***](#_ENREF_22)***]*** | Yes | Yes | NR/Unclear | Yes | NR/Unclear | NR/Unclear | Yes |
| ***Doyle, 2010[***[***23***](#_ENREF_23)***]*** | Yes | Yes | NR/Unclear | Yes | NR/Unclear | NR/Unclear | Yes |
| ***Freysteinson, 2010[***[***24***](#_ENREF_24)***]*** | Yes | Yes | NR/Unclear | NR/Unclear | Yes | NR/Unclear | Yes |
| ***Hanley, 2001[***[***25***](#_ENREF_25)***]*** | Yes | Yes | Yes | Yes | Yes | Yes | Yes |
| ***Jenkins, 2002[***[***26***](#_ENREF_26)***]*** | Yes | Yes | Yes | Yes | Yes | NR/Unclear | Yes |
| ***Johnston, 2008a[***[***27***](#_ENREF_27)***]*** | Yes | Yes | Yes | Yes | NR/Unclear | NR/Unclear | Yes |
| ***Jones, 2006[***[***28***](#_ENREF_28)***]*** | Yes | Yes | Yes | Yes | Yes | Yes | Yes |
| ***Kamps, 1987[***[***29***](#_ENREF_29)***]*** | Yes | Yes | Yes | Yes | Yes | NR/Unclear | Yes |
| ***Kelson 1999[***[***30***](#_ENREF_30)***]*** | Yes | Yes | Yes | Yes | NR/Unclear | NR/Unclear | Yes |
| ***Langston, 2005[***[***31***](#_ENREF_31)***]*** | Yes | Yes | NR/Unclear | NR/Unclear | NR/Unclear | NR/Unclear | Yes |
| ***Leinisch-Dahlke, 2004[***[***32***](#_ENREF_32)***]*** | Yes | Yes | Yes | Yes | NR/Unclear | NR/Unclear | Yes |
| ***Madsen, 2000[***[***33***](#_ENREF_33)***]*** | Yes | Yes | Yes | Yes | NR/Unclear | Yes | Yes |
| ***Meropol, 2003[***[***34***](#_ENREF_34)***]*** | Yes | Yes | Yes | Yes | Yes | Yes | Yes |
| ***Minogue, 2010[***[***35***](#_ENREF_35)***]*** | Yes | Yes | NR/Unclear | NR/Unclear | NR/Unclear | NR/Unclear | Yes |
| ***Noe, 2007[***[***36***](#_ENREF_36)***]*** | Yes | Yes | Yes | Yes | NR/Unclear | Yes | Yes |
| ***Sood, 2009[***[***37***](#_ENREF_37)***]*** | Yes | Yes | Yes | Yes | Yes | Yes | Yes |
| ***Thomas, 1999[***[***38***](#_ENREF_38)***]*** | Yes | Yes | Yes | Yes | Yes | NR/Unclear | Yes |
| ***White, 2005[***[***39***](#_ENREF_39)***]*** | Yes | Yes | NR/Unclear | Yes | NR/Unclear | NR/Unclear | Yes |
| ***Qualitative studies*** | | | | | | | |
| ***Abma, 2005[***[***40***](#_ENREF_40)***]*** | Yes | Yes | NR/Unclear | NR/Unclear | NR/Unclear | NR/Unclear | Yes |
| ***Abma, 2010[***[***41***](#_ENREF_41)***]*** | Yes | Yes | NR/Unclear | Yes | NR/Unclear | NR/Unclear | Yes |
| ***Ågård, 2001[***[***42***](#_ENREF_42)***]*** | Yes | Yes | Yes | Yes | Yes | NR/Unclear | Yes |
| ***Ard, 2005[***[***43***](#_ENREF_43)***]*** | Yes | Yes | Yes | Yes | Yes | Yes | Yes |
| ***Asai, 2004[***[***44***](#_ENREF_44)***]*** | Yes | Yes | NR/Unclear | Yes | Yes | NR/Unclear | Yes |
| ***Australian Government[***[***45***](#_ENREF_45)***]*** | Yes | NR/Unclear | NR/Unclear | NR/Unclear | NR/Unclear | NR/Unclear | Yes |
| ***Brody, 2009[***[***46***](#_ENREF_46)***]*** | Yes | Yes | Yes | Yes | Yes | Yes | Yes |
| ***Campbell, 1998[***[***47***](#_ENREF_47)***]*** | Yes | Yes | NR/Unclear | Yes | Yes | NR/Unclear | Yes |
| ***Carey, 1992[***[***48***](#_ENREF_48)***]*** | Yes | Yes | NR/Unclear | Yes | NR/Unclear | NR/Unclear | Yes |
| ***Carey, 2001[***[***49***](#_ENREF_49)***]*** | Yes | Yes | Yes | Yes | Yes | Yes | Yes |
| ***Caron-Flinterman, 2005a[***[***50***](#_ENREF_50)***]*** | Yes | Yes | Yes | Yes | Yes | Yes | Yes |
| ***Carr, 2003[***[***51***](#_ENREF_51)***]*** | Yes | Yes | Yes | Yes | Yes | Yes | Yes |
| ***Casarett, 2001[***[***52***](#_ENREF_52)***]*** | Yes | Yes | Yes | Yes | Yes | Yes | Yes |
| ***Chenoweth, 1998[***[***53***](#_ENREF_53)***]*** | Yes | Yes | Yes | Yes | Yes | NR/Unclear | Yes |
| ***Corneli, 2007[***[***54***](#_ENREF_54)***]*** | Yes | Yes | Yes | Yes | Yes | Yes | Yes |
| ***Cotterell, 2008[***[***55***](#_ENREF_55)***]*** | Yes | Yes | Yes | Yes | Yes | Yes | Yes |
| ***Cox, 1996[***[***56***](#_ENREF_56)***]*** | Yes | Yes | No | Yes | Yes | No | Yes |
| ***Cox, 2000[***[***57***](#_ENREF_57)***]*** | Yes | Yes | No | Yes | Yes | No | Yes |
| ***Curry, 2006[***[***58***](#_ENREF_58)***]*** | Yes | Yes | Yes | Yes | Yes | No | Yes |
| ***Daly, 2009[***[***59***](#_ENREF_59)***]*** | Yes | Yes | Yes | Yes | Yes | No | No |
| ***Daugherty, 1999[***[***60***](#_ENREF_60)***]*** | Yes | NR/Unclear | NR/Unclear | NR/Unclear | NR/Unclear | NR/Unclear | Yes |
| ***Dellson, 2011[***[***61***](#_ENREF_61)***]*** | Yes | Yes | Yes | Yes | Yes | Yes | Yes |
| ***Dixon-Woods, 2006[***[***62***](#_ENREF_62)***]*** | Yes | Yes | Yes | Yes | Yes | Yes | Yes |
| ***Eng, 2005[***[***63***](#_ENREF_63)***]*** | Yes | Yes | Yes | Yes | Yes | No | Yes |
| ***Featherstone, 1998[***[***64***](#_ENREF_64)***]*** | Yes | Yes | Yes | Yes | Yes | Yes | Yes |
| ***Fern, 2011[***[***65***](#_ENREF_65)***]*** | Yes | Yes | Yes | Yes | Yes | Yes | Yes |
| ***Forbes, 2010[***[***66***](#_ENREF_66)***]*** | Yes | Yes | Yes | Yes | Yes | Yes | Yes |
| ***Garber, 2007[***[***67***](#_ENREF_67)***]*** | Yes | Yes | Yes | Yes | Yes | Yes | Yes |
| ***Gittelsohn, 2010[***[***68***](#_ENREF_68)***]*** | Yes | Yes | Yes | Yes | NR/Unclear | Yes | Yes |
| ***Gooberman, 2008[***[***69***](#_ENREF_69)***]*** | Yes | NR/Unclear | NR/Unclear | NR/Unclear | NR/Unclear | NR/Unclear | Yes |
| ***Harper, 2000[***[***70***](#_ENREF_70)***]*** | Yes | NR/Unclear | NR/Unclear | NR/Unclear | NR/Unclear | NR/Unclear | Yes |
| ***Higgins, 2001[***[***71***](#_ENREF_71)***]*** | Yes | Yes | Yes | Yes | No | No | No |
| ***Hsu, 2010[***[***72***](#_ENREF_72)***]*** | Yes | Yes | NR/Unclear | Yes | NR/Unclear | Yes | Yes |
| ***Hutchison, 1998[***[***73***](#_ENREF_73)***]*** | Yes | Yes | Yes | Yes | Yes | Yes | Yes |
| ***Irani, 2010[***[***74***](#_ENREF_74)***]*** | Yes | Yes | Yes | Yes | Yes | Yes | Yes |
| ***Jenkins, 2005[***[***75***](#_ENREF_75)***]*** | Yes | Yes | Yes | Yes | Yes | Yes | Yes |
| ***Jinks, 2009[***[***76***](#_ENREF_76)***]*** | Yes | Yes | No | Yes | Yes | Yes | Yes |
| ***Karlawish, 2008[***[***77***](#_ENREF_77)***]*** | Yes | Yes | Yes | Yes | Yes | Yes | Yes |
| ***Karmaliani, 2009[***[***78***](#_ENREF_78)***]*** | Yes | Yes | Yes | Yes | NR/Unclear | Yes | Yes |
| ***Kelly, 2005[***[***79***](#_ENREF_79)***]*** | Yes | NR/Unclear | NR/Unclear | NR/Unclear | NR/Unclear | NR/Unclear | Yes |
| ***King, 2009[***[***80***](#_ENREF_80)***]*** | Yes | Yes | Yes | Yes | Yes | Yes | Yes |
| ***Kirwan, 2003[***[***81***](#_ENREF_81)***]*** | Yes | Yes | Yes | Yes | Yes | Yes | Yes |
| ***Kirwan, 2005[***[***82***](#_ENREF_82)***]*** | Yes | Yes | Yes | Yes | Yes | Yes | Yes |
| ***Lammers, 2004[***[***83***](#_ENREF_83)***]*** | Yes | Yes | Yes | NR/Unclear | Yes | No | Yes |
| ***Lavender, 2009[***[***84***](#_ENREF_84)***]*** | Yes | Yes | Yes | Yes | Yes | Yes | Yes |
| ***Lindenmeyer, 2007[***[***85***](#_ENREF_85)***]*** | Yes | Yes | NR/Unclear | Yes | Yes | Yes | Yes |
| ***Llewellyn-Thomas, 1989[***[***86***](#_ENREF_86)***]*** | Yes | Yes | Yes | Yes | Yes | Yes | Yes |
| ***MacKinnon, 2010[***[***87***](#_ENREF_87)***]*** | Yes | Yes | Yes | Yes | Yes | Yes | Yes |
| ***Manson, 2004[***[***88***](#_ENREF_88)***]*** | Yes | NR/Unclear | Yes | NR/Unclear | Yes | NR/Unclear | Yes |
| ***Martin, 2009[***[***89***](#_ENREF_89)***]*** | Yes | Yes | Yes | Yes | Yes | Yes | Yes |
| ***Maslin-Prothero, 2003[***[***90***](#_ENREF_90)***]*** | Yes | NR/Unclear | NR/Unclear | NR/Unclear | NR/Unclear | NR/Unclear | Yes |
| ***Mastwyk, 2002[***[***91***](#_ENREF_91)***]*** | Yes | Yes | Yes | Yes | Yes | Yes | Yes |
| ***McQuiston, 2005[***[***92***](#_ENREF_92)***]*** | Yes | Yes | NR/Unclear | Yes | NR/Unclear | Yes | Yes |
| ***Mease , 2007[***[***93***](#_ENREF_93)***]*** | Yes | Yes | Yes | Yes | NR | No | Yes |
| ***Medd, 2005[***[***94***](#_ENREF_94)***]*** | Yes | Yes | Yes | Yes | Yes | No | Yes |
| ***Milewa, 2008[***[***95***](#_ENREF_95)***]*** | Yes | NR/Unclear | NR/Unclear | NR/Unclear | NR/Unclear | NR/Unclear | Yes |
| ***Mills, 2003[***[***96***](#_ENREF_96)***]*** | Yes | Yes | Yes | Yes | Yes | Yes | Yes |
| ***Minkler, 2002[***[***97***](#_ENREF_97)***]*** | Yes | Yes | Yes | Yes | Yes | Yes | Yes |
| ***Moreno-Black, 2004[***[***98***](#_ENREF_98)***]*** | Yes | Yes | Yes | Yes | Yes | Yes | Yes |
| ***Morin, 2008[***[***99***](#_ENREF_99)***]*** | Yes | Yes | Yes | Yes | NR/Unclear | Yes | Yes |
| ***Murad, 2011[***[***100***](#_ENREF_100)***]*** | Yes | Yes | Yes | Yes | Yes | Yes | Yes |
| ***Nair, 2004[***[***101***](#_ENREF_101)***]*** | Yes | Yes | Yes | Yes | Yes | Yes | Yes |
| ***Ntshanga, 2010[***[***102***](#_ENREF_102)***]*** | Yes | NR/Unclear | NR/Unclear | NR/Unclear | NR/Unclear | NR/Unclear | Yes |
| ***Ong, 2003[***[***103***](#_ENREF_103)***]*** | Yes | Yes | Yes | Yes | NR/Unclear | NR/Unclear | Yes |
| ***Paul, 2011[***[***104***](#_ENREF_104)***]*** | Yes | Yes | Yes | Yes | Yes | Yes | Yes |
| ***Plumb, 2008[***[***105***](#_ENREF_105)***]*** | Yes | Yes | Yes | Yes | Yes | Yes | Yes |
| ***Read, 2009[***[***106***](#_ENREF_106)***]*** | Yes | Yes | Yes | Yes | Yes | Yes | Yes |
| ***Reddy, 2010[***[***107***](#_ENREF_107)***]*** | Yes | Yes | Yes | Yes | Yes | Yes | Yes |
| ***Redwood, 2010[***[***108***](#_ENREF_108)***]*** | Yes | Yes | Yes | Yes | Yes | No | Yes |
| ***Richards, 2002[***[***109***](#_ENREF_109)***]*** | Yes | Yes | Yes | NR/Unclear | NR/Unclear | NR/Unclear | Yes |
| ***Roberts, 2000[***[***110***](#_ENREF_110)***]*** | Yes | NR/ Unclear | Yes | Yes | Yes | Yes | Yes |
| ***Roberts, 2002[***[***111***](#_ENREF_111)***]*** | Yes | NR/ Unclear | Yes | Yes | Yes | Yes | Yes |
| ***Roberts, 2004[***[***112***](#_ENREF_112)***]*** | Yes | Yes | Yes | Yes | Yes | Yes | Yes |
| ***Roe, 1995[***[***113***](#_ENREF_113)***]*** | Yes | Yes | Yes | Yes | Yes | NR/Unclear | Yes |
| ***Rogers, 1994[***[***114***](#_ENREF_114)***]*** | Yes | Yes | NR/Unclear | Yes | NR/Unclear | NR/Unclear | Yes |
| ***Rosen, 2007[***[***115***](#_ENREF_115)***]*** | Yes | NR/ Unclear | Yes | NR/ Unclear | Yes | Yes | Yes |
| ***Ross, 2005[***[***116***](#_ENREF_116)***]*** | Yes | NR/ Unclear | Yes | Yes | Yes | NR/Unclear | Yes |
| ***Saurbrey, 1984[***[***117***](#_ENREF_117)***]*** | Yes | Yes | Yes | Yes | Yes | Yes | Yes |
| ***Savage, 2006[***[***118***](#_ENREF_118)***]*** | Yes | NR/ Unclear | Yes | Yes | Yes | No | Yes |
| ***Sayers, 2009[***[***119***](#_ENREF_119)***]*** | Yes | Yes | Yes | No | NR/ Unclear | No | Yes |
| ***Serrano-Aguilar, 2009[***[***120***](#_ENREF_120)***]*** | Yes | NR/ Unclear | Yes | Yes | Yes | Yes | Yes |
| ***Shilling, 2011[***[***121***](#_ENREF_121)***]*** | Yes | Yes | Yes | Yes | Yes | Yes | Yes |
| ***Slomka, 2008[***[***122***](#_ENREF_122)***]*** | Yes | Yes | Yes | Yes | Yes | Yes | Yes |
| ***Smith, 2007[***[***123***](#_ENREF_123)***]*** | Yes | No | Yes | NR/ Unclear | NR/ Unclear | No | NR/ Unclear |
| ***Snowdon, 2006[***[***124***](#_ENREF_124)***]*** | Yes | Yes | Yes | Yes | Yes | Yes | Yes |
| ***Snowdon, 1997[***[***125***](#_ENREF_125)***]*** | Yes | Yes | Yes | Yes | Yes | NR/Unclear | Yes |
| ***Staniszewska, 2007[***[***126***](#_ENREF_126)***]*** | Yes | Yes | NR/Unclear | Yes | NR/Unclear | NR/Unclear | Yes |
| ***Staniszewska, 2011[***[***127***](#_ENREF_127)***]*** | Yes | NR/Unclear | NR/Unclear | NR/Unclear | NR/Unclear | NR/Unclear | Yes |
| ***Stewart, 2006[***[***128***](#_ENREF_128)***]*** | Yes | NR/Unclear | NR/Unclear | NR/Unclear | NR/Unclear | NR/Unclear | Yes |
| ***Stewart, 2009[***[***129***](#_ENREF_129)***]*** | Yes | NR/ Unclear | Yes | Yes | Yes | Yes | Yes |
| ***Stirman, 2010[***[***130***](#_ENREF_130)***]*** | Yes | NR/ Unclear | Yes | Yes | NR/ Unclear | Yes | Yes |
| ***Sugarman,1998[***[***131***](#_ENREF_131)***]*** | Yes | Yes | Yes | Yes | Yes | Yes | Yes |
| ***Sullivan, 2005[***[***132***](#_ENREF_132)***]*** | Yes | Yes | Yes | Yes | Yes | Yes | Yes |
| ***Thurston, 2005[***[***133***](#_ENREF_133)***]*** | Yes | Yes | Yes | Yes | Yes | Yes | Yes |
| ***Timotijevic, 2007[***[***134***](#_ENREF_134)***]*** | Yes | Yes | Yes | Yes | Yes | Yes | Yes |
| ***Tischler, 2010[***[***135***](#_ENREF_135)***]*** | Yes | Yes | Yes | Yes | Yes | Yes | Yes |
| ***Tobin, 2002[***[***136***](#_ENREF_136)***]*** | Yes | Yes | Yes | Yes | Yes | Yes | Yes |
| ***Van Olphen, 2009[***[***137***](#_ENREF_137)***]*** | Yes | Yes | Yes | Yes | NR/Unclear | NR/Unclear | Yes |
| ***van Staa, 2010[***[***138***](#_ENREF_138)***]*** | Yes | Yes | Yes | Yes | Yes | Yes | Yes |
| ***Wersch, 2001[***[***139***](#_ENREF_139)***]*** | Yes | NR/Unclear | NR/Unclear | NR/Unclear | NR/Unclear | NR/Unclear | Yes |
| ***White, 2008[***[***140***](#_ENREF_140)***]*** | Yes | Yes | Yes | Yes | Yes | Yes | Yes |
| ***Wright, 1997[***[***141***](#_ENREF_141)***]*** | Yes | Yes | Yes | Yes | Yes | Yes | Yes |
| ***Zullino, 2003[***[***142***](#_ENREF_142)***]*** | Yes | Yes | Yes | Yes | Yes | Yes | Yes |

*NR: not reported.

### Table S4: Initiatives and patient organizations identified by the environmental scan:

| ***Title/source/***  ***author/origin*** | ***URL*** | ***Content description/conclusions*** |
| --- | --- | --- |
| ***Forum: Bringing The Patient Voice Into Research***  ***Youtube.com***  ***PatientsLikeMe*** | http://www.youtube.com/watch?v=pn5jb-eIf2E | The Website encourages disease specific forums in which patients share their symptoms and responses to treatment, encouraging patient initiated research which sets the agenda and provides the pilot data for more rigorous research. |
| ***Organization: The Association of Cancer Online Resources, Inc. (ACOR)***  ***USA*** | _ | ACOR is a non-profit organization incorporated in New York that offers information and support through its integrated system of online discussion groups. It offers access to 159 mailing lists that provide support, information, and community to everyone affected by cancer and related disorders. ACOR creates specific websites and also hosts a growing number of websites, created by:   - Patients for patients and caregivers. - Cancer Advocacy Organizations, many of which were created directly from the membership of an ACOR mailing list. - Professional Organizations.   The site educate patients and empower them to participate in research (clinical trial FAQ; guide to trial terminology, access to investigational drugs and a guide to find a clinical trial) |
| ***Organization: The Patients Voice by Healthcare Landscape***  ***UK*** | http://www.thepatientsvoice.org/ | The Patients Voice is run by company that invites patients, their friends, family and carers to participate in market research. Members can interact with each other through a social network forum called the Icare Café. |
| ***White Paper: Patient and Public Involvement (PPI) in the Research Process***  ***Nathan Shippee, Anna Johnson, Victor Montori (Mayo Clinic, USA)*** | _ | The paper describes a framework for involving patients in research and defines the potential areas and roles for this involvement |
| ***Blog: New Voices for Research*** | http://newvoicesforresearch.blogspot.com/ | A blog that allows direct communication between early career researchers so that they become advocates for communities. No clear description of methods for patient engagement |
| ***Organization: The Lupus Research Institute*** | http://www.lupusresearchinstitute.org/news/discoveries/09_advocacy | A patient was nominated to serve as a “consumer reviewer” for scientific lupus proposals submitted to the Department of Defense “Peer Reviewed Medical Research Program.” That year, the research program received $50 million in appropriations to fund research in 21 research topic areas—including lupus. |
| ***Network: Community of practice in ecohealth***  ***Canada*** | http://www.copeh-canada.org/index_en.php | A network made up of individuals and organizations that share common concerns or interest in ecohealth. Their methods could be a model extrapolated to biomedical research.  Their vision:  - to build the community’s foundation with the support of the three initiating university’s expertise on the Ecohealth approach; an expertise that hinges on research projects, education, and development work with Latin American, African and Asian partners;  -to support the progressive development of the community of practice through the participation of colleagues, partners and collaborators interested in the ecosystem approach to health;  -to support the development of regional groups and sustainable studies that aim to inform research, education and public policy about the contributions and ideas that result from ecosystem approaches to health |
| ***Organization: National Institutes for Health Research (NIHR)***  ***UK*** | http://www.nihr.ac.uk/awareness/Pages/default.aspx | The organization vision is that Involving patients and members of the public in research can lead to better research, clearer outcomes, and faster uptake of new evidence.  Goals:   - Set research priorities - Identify the important questions that health and social care research needs to answer - Give their views on research proposals alongside clinicians, methodologists, scientists, and public health and other professionals - Help assess proposals for funding - Take part in clinical trials and other health and social care research studies, not just as subjects but as active partners in the research process - Publicize the results. - Available structures to fulfill goals: - All the NIHR’s Research Programmes actively engage patients and the public in all stages of research. The NIHR coordinating centres recruit members of the public to help in the commissioning and reviewing of NIHR research proposals. - The funding of INVOLVE which promotes active public participation in NHS, public health and social care research to improve the way that research is prioritised, commissioned, undertaken, communicated and used. - The establishment of the Clinical Research Network across England to increase the number of people recruited onto clinical trials and to the validity of their results. |
| ***Organization: INVOLVE***  ***UK*** | http://www.invo.org.uk/ | A national advisory group created and funded by the National Institute of Health Research and supports greater public involvement in public health and social care research. Thirty members help to identify issues that need to be addressed by INVOLVE and also to disseminate new ideas on policy and practice.  Vision:  -maximize the opportunities for public involvement  -learn and share knowledge and experiences of public involvement  -support inclusion, diversity and equity and the active involvement in research of groups and individuals who are often excluded from research  Rich site with a database of published and unpublished research projects in the field of health, public health and social care that have or plan to actively involve members of the public as partners in the research process. |
| ***Organization: James Lind Alliance***  ***UK*** | http://www.lindalliance.org | The alliance facilitates Priority Setting Partnerships by bringing patients, carers and clinicians together to identify and prioritize for research the treatment uncertainties which they agree are the most important.  Vision:  -addressing uncertainties about the effects of treatments should become accepted as a much more routine part of clinical practice  -patients, carers and clinicians should work together to agree which, among those uncertainties, matter most and thus deserve priority attention  Workflow:  The alliance facilitates the collaboration between an organization and the patients and helps create a Steering Group, which comprises both clinical and patient representation who sign a Protocol setting out their aims for the Partnership, and their agreed commitments.  The key components of a priority setting process are:   - - engaging with patients, carers and clinicians to participate in the Partnership   - collecting patients', carers' and clinicians' treatment uncertainties, then checking them   - reviewing existing research recommendations to identify uncertainty   - working with patients, carers and clinicians to prioritise the uncertainties, and agreeing a ranked top 10 questions for research to address   Includes bibliography of research reports about patients', clinicians' and researchers' priorities for new research (identified through a fairly rigorous scoping study) |
| ***Book: Patients, the public and priorities in healthcare,*** *Edited by Peter Littlejohns and Michael Rawlins.* ***Radcliffe, 2009.*** | http://www.lindalliance.org/Publications.asp | Focus on public involvement in agenda setting agenda setting |
| ***Organization: Patient Voices Network*** | http://www.patientvoices.ca/ | At the core of the Patient Voices Network is the belief that patients affected by a health care decision should be involved in the process of reaching that decision. |
| ***Organization: International Association of Public Participation*** | http://www.iap2.org/ | An association that advocates for public participation as means to involve those who are affected by a decision in the decision-making process and it communicates to participants how their input affects the decision. The association provides training and professional development (certificate in public participation). The focus is not research and not healthcare. |
| ***Organization: National Cancer Institute***  ***USA*** | http://outcomes.cancer.gov/areas/pcc/communication/monograph.html | The focus is on Patient-Centered Communication in Cancer Care. No clear descriptions of methods of engagement in research |
| ***Organization: the Community Impacts  of Research Oriented Partnerships  (CIROP) Measure***  ***Canada*** | http://impactmeasure.org/about.htm | Researchers from five community-university research partnerships from Ontario, Canada, joined together to develop a reliable and valid survey to measure the community impacts of research partnerships between universities and community agencies.  **The** CIROP Measure **is a 33-item, generic measure of community members' perceptions of the impact of research partnerships. It** can be used to:   1. assess the effectiveness of knowledge sharing approaches, 2. determine the most influential activities of partnerships, 3. determine structural characteristics of partnerships associated with various types of impact, and 4. Demonstrate accountability to funding bodies. |
| ***Organization: Civic Change (Pew Partnership for Change)***  ***USA*** | http://www.pew-partnership.org/whoweare.html | An example of community-university Partnership. This civic research organization provides consulting and program support to communities, governments, foundations, and nonprofit agencies to help clients identify and implement solutions and strategies crucial to making communities stronger. |
| ***Organization: Health Issues Centre***  ***Australia*** | http://www.healthissuescentre.org.au/subjects/list-library-subject.chtml?subject=44 | An independent, not-for-profit organization that promotes equity and consumer perspectives in the Australian health system. Its mission is to improve the health outcomes especially the disadvantaged.  Activities: -policy analysis and advocacy from consumer and equity perspectives  -consumer-focused research  -promoting and supporting consumer participation  -disseminating information.  Model: The organizations is approached by systems/clients in need of consumer perspective and link them to database of available consumers |
| ***A Model Framework for***  ***Consumer and Community Participation in Health and Medical Research. National Health and Medical Research Council. Commonwealth of Australia 2005.***  ***Australia*** | http://www.healthissuescentre.org.au/documents/items/2011/01/360783-upload-00001.pdf | A detailed model is described in 57 page document providing a framework for consumer and community participation in health and medical research  The model describes the current practice and the proposed one (involving the public) in every step of the research process. |
| ***Workshop: Consumer and Community Involvement in Research, a course for researchers.***  ***University of Western Australia workshop*** | http://www.sph.uwa.edu.au/courses/winter-spring-summer-school/research-involvement | This workshop aimed at increasing awareness of the contribution consumers and community members can make to research; identifying and addressing the barriers to consumer and community participation; developing understanding and skills on the 'how and why' for implementing consumer and community participation; providing information about resources and other sources of help to support consumer and community participation). December 2011 |
| ***Database: PubMed Health***  ***USA*** | http://www.ncbi.nlm.nih.gov/pubmedhealth/ | PubMed Health specializes in reviews of clinical effectiveness research, with easy-to-read summaries for consumers as well as full technical reports. PubMed Health is a service provided by the National Center for Biotechnology Information (NCBI) at the U.S. National Library of Medicine (NLM). This represents a good tool for patients to become more informed. No recommended methods of engagement; however. |
| ***Center: Center for Shared Decision Making***  ***USA*** | http://patients.dartmouth-hitchcock.org/shared_decision_making.html | Indirectly relevant resource. The focus is on shared decision making in clinical context. One-on-one counseling sessions for any medical condition are provided along with a Decision Aid Library of helpful videotapes, audiotapes, booklets, CD-ROMs, and websites and a Health Care Decision Guide |
| ***Center: Mayo Clinic Shared Decision Making National Resource Center***  ***USA*** | http://shareddecisions.mayoclinic.org/ | Potentially relevant resource. The focus is on shared decision making in clinical context. However, trials of decision aids are frequently conducted with heavy input from patients and designers, as well as an advisory patient group. Mission:  Developing and evaluating patient decision aids  Contributing to defining international decision aid standards  Defining high performance organizations using shared decision making  Educating and training care providers in communication techniques  Adopting and using patient decision aids at the point of care  Contributing to statewide implementation efforts  Certifying patient decision aids through collaboration with external partners |
| ***Center: Society for Participator Medicine***  ***USA*** | http://participatorymedicine.org/ | Potentially relevant resource. The focus is on shared decision making in clinical context. It is a cooperative model of health care that encourages and expects active involvement by all connected parties (patients, caregivers, healthcare professionals, etc.) as integral to the full continuum of care. The ‘participatory’ concept may also be applied to fitness, nutrition, mental health, end-of-life care, and all issues broadly related to an individual’s health. |
| ***Center: CARE: Community Alliance for Research and Engagement. Yale Center for Clinical Investigation.***  ***USA*** | http://care.yale.edu/index.aspx | The alliance fosters rigorous community-based research and to translate scientific breakthroughs into practical benefits for residents of New Haven. In partnership with local health centers and hospitals, the city of New Haven, community organizations, businesses, faith communities, and other parts of Yale University, this School of Public Health program brings together diverse people and organizations to improve health in the city of New Haven. Its cornerstone project, Community Interventions for Health (CIH), is an international collaborative of the Oxford Health Alliance that addresses chronic disease risk factors— unhealthy diet, physical inactivity and tobacco use— through policy and structural initiatives and health promotion programs. |
| ***Organization: Healthcare Financial Management Association*** | [http://www.hfma.org](http://www.hfma.org/) | The focus is mostly from management/financial point of view |
| ***Center: Institute for Participatory Action Research & Design*** | http://web.gc.cuny.edu/che/start.htm | The website describes several projects with clear patient engagement. The projects seek to reveal theoretically and empirically the contours of injustice and resistance and challenge the traditional conceptions of "expert knowledge. The focus is on youth. |
| ***Center: Center for Participatory Action Research*** | http://cadres.pepperdine.edu/ccar/define.html | **The website describes the goals of Action Research as:** The improvement of professional practice through continual learning and progressive problem solving;  A deep understanding of practice and the development of a well specified theory of action;  An improvement in the community in which one's practice is embedded through participatory research.  A model for learning cycles and feedback is described. |
| ***Website: Learning for sustainability*** | http://learningforsustainability.net/research/action_research.php | Numerous Participatory Action Research projects and methodologies are described. A relevant publication (Stakeholder Participation for Environmental Management: A Literature Review) points to the need to focus on participation as a process. It then identifies a number of best practice features from the literature. Finally, it argues that to overcome many of its limitations, stakeholder participation must be institutionalized, creating organizational cultures that can facilitate processes where goals are negotiated and outcomes are necessarily uncertain. The paper acknowledges that seen in this light, participatory processes may seem very risky, but there is growing evidence that if well designed, these perceived risks may be well worth taking. |
| ***National Resource Centre for Consumer Participation*** | http://www.healthissuescentre.org.au/subjects/list-library-subject.chtml?subject=44 | This website includes major policy documents and history of consumer participation. A Clearinghouse for information on consumer feedback and participation methodologies |
| ***Book: Avard D etal. Research Ethics Boards and Challenges for Public Participation. Health Law Review, 2009;17(2-3) 66-72*** | E-book: http://www.amazon.com/Research-ethics-boards-challenges-participation/dp/B002F1RB2W | Ethical focus. |
| ***Organization: Health Quality Improvement Partnership***  ***UK*** | http://www.hqip.org.uk/patient-and-public-engagement-2/ | The organization was established in 2008 to promote quality in healthcare. They consider effective patient and public involvement/engagement in clinical audit to be fundamental to good quality improvement practice. The website contains templates and case studies |
| ***Project: Patient Partner***  ***Europe*** | www.patientpartner-europe.eu | A three year project funded by the European Commission and ended in 2011 and aimed to promote the role of patient organizations in the clinical trials context PatientPartner was based on the belief that involving patient organizations as equal partners at all stages of clinical trials contributes to research that is better adjusted to the real needs of patients.  The study looked closely at the part that patient organizations play and are willing to play in clinical trials and also focused its attention on clinical trials with children, the use of biobanks and ethical issues.  The main objectives of the project were;  • To identify best practices of patient organization’s active involvement in clinical research in Europe. • To facilitate the dialogue between all involved stakeholders in clinical research as to how to establish this involvement and integral part of clinical research • Develop guidance material to be used by all stakeholders in order to facilitate the future partnerships in clinical research between patient organizations, sponsors and investigators. |
| ***NHS - Patient Involvement Toolkit*** | www.rcn.org.uk/?a=56801 | Very relevant guide (toolkit) that describes the principles of patient engagements—mostly based on hypothesis and not empirical evidence |
| ***Health Canada Policy Toolkit for Public Involvement in Decision Making*** | http://www.hc-sc.gc.ca/ahc-asc/pubs/_public-consult/2000decision/index-eng.php | Case studies are available describing various projects that included patient participation, less focused on research |
| ***Center for Patient Partnerships. University of Wisconsin-Madison***  ***USA*** | http://www.patientpartnerships.org/research/ | The Center for Patient Partnerships directly documents the impact of advocacy services on patients through evaluation, field research, and synthesis of existing empirical studies.  Ongoing research activities:  State Consumer Assistance/Patient Support Policy: A Comparative Analysis  Comparative Analysis of Advocacy and Other Support Services  The Social Compact for Advancing Team-Based Care  Survivorship Care Planning  Consumer Engagement in Health Systems Quality Improvement |
| ***CAPOIRA: Increasing patient involvement in research activities***  ***Europe*** | http://www.eurordis.org/content/capoira-increasing-patient-involvement-research-activities | The CAPOIRA project, funded by the European Commission, facilitates the creation of structural links to bring civil society and the scientific community closer together. The main idea is to foster the participation of patient organizations in research activities by increasing their knowledge, skills and capabilities in the two areas of clinical trials (private or public; at national or European level) and EU-funded health research projects. Training is offered to strengthen the capacity of rare disease patients’ representatives and empower patients’ representatives to advocate effectively for rare diseases at both the local and EU level. Disease specific forums are available. The focus is on rare diseases and orphan drugs. |
| ***Organization: National Association for Patient Participation*** | <http://www.napp.org.uk/> | The association promotes the proactive engagement of patients through ‘Patient Reference Groups’ focusing mainly on community engagement and health. Research engagement is limited to policy and agenda setting. Tools used are mainly surveys. |
| ***Presentation: Jo Brett, Sophie Staniszewska, Sandy Herron-Marx, Kate Seers, Helen Bayliss, Carole Mockford School of Health and Social Studies, Warwick University*** | www.rcn.org.uk/__data/assets/pdf_file/0010/380665/2011_RCN | Presentation of framework for patient and public involvement (PPI). Highlights challenges and the need for better reporting of PPI in published journal articles and study reports. |
| ***Toolkit: Working With Practices and Communitie. Washington's Institute for Translational Health Sciences Clinical and Translational Sciences.*** | http://www.researchtoolkit.org/home/developing-proposals/working-with-practices-and-communities.html | Partnership-driven Resources to IMprove and Enhance Research (PRIMER).  The PRIMER project was funded through an administrative supplement to the University of Washington's Institute for Translational Health Sciences Clinical and Translational Sciences Award (CTSA) UL1 RR025014 from the NIH National Center for Research Resources. |
| ***Partnership Self-Assessment tool from the Center for the Advancement of Collaborative Strategies in Health. From the Center for the Advancement of Collaborative Strategies in Health, Division of Public Health, New York Academy of Medicine.*** | http://partnershiptool.net/ | The Center for the Advancement of Collaborative Strategies in Health has developed ideas and tools to enhance partnerships, including an adaptable tool to measure strength and cohesion in a given group. The Partnership Self-Assessment tool, scoring instructions and a coordinator's guide are all freely available for download, along with a published article on the concepts underlying synergy. |
| ***Patient group: Diabetes Advisory Group. Mayo Clinic, Rochester.***  ***USA*** | http://shareddecisions.mayoclinic.org/stakeholders/diabetes-advisory-group/ | A group of community members with diabetes in the Rochester, Minnesota, and surrounding area.  This advisory group has been meeting with Mayo Clinic researchers on a monthly basis for the past 7 years to provide feedback on research proposals, participant recruitment materials, surveys, and all areas of proposed and existing research.  The members view research through a patient’s perspective, which assists in detecting potential barriers and contributes to effective and meaningful research. Members contribute their personal time and practical experiences in living with diabetes, and through their involvement, researchers remain connected to the real world of the patients that will be impacted |
| ***Patient group: One Voice Patient & Family Advisory Council. Mayo Clinic, Rochester.***  ***USA*** | http://shareddecisions.mayoclinic.org/stakeholders/one-voice-advisory-council/ | This advisory council is created by patients and their families to contribute to the design and operations of cardiovascular clinical services and research. They publish a quarterly newsletter written for and by patients and families. |
| ***Organization: The Parkinson Pipeline Project*** | http://www.pdpipeline.org/ | A grassroots group of advocates whose goal is to provide the patient perspective in the treatment development process of Parkinson disease. The group develops a cadre of well-informed PD patient consultants.  -Promotes policies that accelerate the evaluation, approval of safe, effective, and timely new treatments by providing the unique patient perspectives to industry sponsors and investors, clinical scientists, and government regulatory agencies.  -Maintains a comprehensive database of information Tracks the progress of new therapies and diagnostics  -Recruits, trains, provides peer supervision, and up-to-date information on the views of grass roots opinion leaders and clinical trial participants to represent authentic patient interests in the regulatory processes of the FDA in cooperation with the FDA's Office of Special Health Issues.  -Increases medical provider and patient awareness  -Advocates for the rights of clinical trial participants in relation to sponsors as well as researchers and offers participants the confidence of peer support to address grievances with researchers or sponsors |

**References:**

1. Boote J, Baird W, Beecroft C: **Public involvement at the design stage of primary health research: a narrative review of case examples**. 2010, **95**(1):10-23.

2. Brett J, Staniszewska S, Mockford C, Seers K, Herron-Marx S, Bayliss H: **The PIRICOM Study: A systematic review of the conceptualisation, measurement, impact and outcomes of patients and public involvement in health and social care research.** In*.* Warwick; 2010.

3. Hussain-Gambles M, Leese B, Atkin K, Brown J, Mason S, Tovey P: **Involving South Asian patients in clinical trials**. 2004, **8**(42):iii, 1-109.

4. Legare F, Boivin A, van der Weijden T, Pakenham C, Burgers J, Legare J, St-Jacques S, Gagnon S: **Patient and public involvement in clinical practice guidelines: a knowledge synthesis of existing programs**. *Med Decis Making* 2011, **31**(6):E45-74.

5. Mockford C, Staniszewska S, Griffiths F, Herron-Marx S: **The impact of patient and public involvement on UK NHS health care: a systematic review**. *Int J Qual Health Care* 2012, **24**(1):28-38.

6. Nilsen ES, Myrhaug HT, Johansen M, Oliver S, Oxman AD: **Methods of consumer involvement in developing healthcare policy and research, clinical practice guidelines and patient information material**. 2006, **3**:CD004563.

7. Oliver S, Clarke-Jones L, Rees R, Milne R, Buchanan P, Gabbay J, Gyte G, Oakley A, Stein K: **Involving consumers in research and development agenda setting for the NHS: developing an evidence-based approach**. 2004, **8**(15):1-148.

8. Stewart RJ, Caird J, Oliver K, Oliver S: **Patients' and clinicians' research priorities**. 2011, **14**(4):439-448.

9. Atkinson NL, Massett HA, Mylks C, McCormack LA, Kish-Doto J, Hesse BW, Wang MQ: **Assessing the impact of user-centered research on a clinical trial eHealth tool via counterbalanced research design**. 2011, **18**(1):24-31.

10. Daugherty C, Ratain MJ, Grochowski E, Stocking C, Kodish E, Mick R, Siegler M: **Perceptions of cancer patients and their physicians involved in phase I trials.[Erratum appears in J Clin Oncol 1995 Sep;13(9):2476]**. 1995, **13**(5):1062-1072.

11. Edwards V, Wyatt K, Logan S, Britten N: **Consulting parents about the design of a randomized controlled trial of osteopathy for children with cerebral palsy**. 2011, **14**(4):429-438.

12. Koops L, Lindley RI: **Thrombolysis for acute ischaemic stroke: consumer involvement in design of new randomised controlled trial**. 2002, **325**(7361):415.

13. Marsden J, Bradburn J, Clinical TCAGf, Macmillan CLJ: **Patient and clinician collaboration in the design of a national randomized breast cancer trial**. 2004, **7**(1):6-17.

14. Shagi C, Vallely A, Kasindi S, Chiduo B, Desmond N, Soteli S, Kavit N, Vallely L, Lees S, Hayes R *et al*: **A model for community representation and participation in HIV prevention trials among women who engage in transactional sex in Africa**. 2008, **20**(9):1039-1049.

15. Swartz LJ, Callahan KA, Butz AM, Rand CS, Kanchanaraksa S, Diette GB, Krishnan JA, Breysse PN, Buckley TJ, Mosley AM *et al*: **Methods and issues in conducting a community-based environmental randomized trial**. 2004, **95**(2):156-165.

16. Andejeski Y, Bisceglio IT, Dickersin K, Johnson JE, Robinson SI, Smith HS, Visco FM, Rich IM: **Quantitative impact of including consumers in the scientific review of breast cancer research proposals**. 2002, **11**(4):379-388.

17. Bigrigg A: **The patient's perspective of commercial clinical trials**. 1999, **4**(3):128-134.

18. Caron-Flinterman JF, Broerse JE, Teerling J, Bunders JF: **Patients' priorities concerning health research: the case of asthma and COPD research in the Netherlands**. 2005, **8**(3):253-263.

19. Cashman SB, Adeky S, Allen AJ, Iii, Corburn J, Israel BA, Montano J, Rafelito A, Rhodes SD, Swanston S *et al*: **The power and the promise: working with communities to analyze data, interpret findings, and get to outcomes**. 2008, **98**(8):1407-1417.

20. Crowe JL, Keifer MC, Salazar MK: **Striving to provide opportunities for farm worker community participation in research**. 2008, **14**(2):205-219.

21. Davison BJ, So A, Goldenberg SL, Berkowitz J, Gleave ME: **Measurement of factors influencing the participation of patients with prostate cancer in clinical trials: a Canadian perspective**. 2008, **101**(8):982-987.

22. Dencker SJ, Boulougouris J, Greist J: **Research values and priorities rated by psychiatric patients**. 1986, **74**(Suppl 331):114-119.

23. Doyle M, Timonen V: **Lessons from a community-based participatory research project: older people's and researcher's reflections**. 2010, **32**(2):244-263.

24. Freysteinson WM: **The ethical community consultation model as preparation for nursing research: a case study**. 2010, **17**(6):749-758.

25. Hanley B, Truesdale A, King A, Elbourne D, Chalmers I: **Involving consumers in designing, conducting, and interpreting randomised controlled trials: questionnaire survey**. 2001, **322**(7285):519-523.

26. Jenkins V, Leach L, Fallowfield L, Nicholls K, Newsham A: **Describing randomisation: patients' and the public's preferences compared with clinicians' practice**. 2002, **87**(8):854-858.

27. Johnston B, Forbat L, Hubbard G: **Involving and engaging patients in cancer and palliative care research: workshop presentation**. 2008, **14**(11):554-557.

28. Jones JM, Nyhof-Young J, Moric J, Friedman A, Wells W, Catton P: **Identifying motivations and barriers to patient participation in clinical trials**. 2006, **21**(4):237-242.

29. Kamps WA, Akkerboom JC, Kingma A, Humphrey GB: **Experimental chemotherapy in children with cancer--a parent's view**. 1987, **4**(2):117-124.

30. Kelson MC: **Consumer collaboration, patient-defined outcomes and the preparation of Cochrane Reviews**. 1999, **2**(2):129-135.

31. Langston AL, McCallum M, Campbell MK, Robertson C, Ralston SH: **An integrated approach to consumer representation and involvement in a multicentre randomized controlled trial**. 2005, **2**(1):80-87.

32. Leinisch-Dahlke E, Akova-Ozturk E, Bertheau U, Isberner I, Evers S, May A: **Patient preference in clinical trials for headache medication: the patient's view**. 2004, **24**(5):347-355.

33. Madsen SM, Holm S, Riis P: **The extent of written trial information: preferences among potential and actual trial subjects**. 2000, **159**:13-18.

34. Meropol NJ, Weinfurt KP, Burnett CB, Balshem A, Benson AB, rd, Castel L, Corbett S, Diefenbach M, Gaskin D *et al*: **Perceptions of patients and physicians regarding phase I cancer clinical trials: implications for physician-patient communication**. 2003, **21**(13):2589-2596.

35. Minogue V, Girdlestone J: **Building capacity for service user and carer involvement in research: the implications and impact of best research for best health**. 2010, **23**(4):422-435.

36. Noe TD, Manson SM, Croy C, McGough H, Henderson JA, Buchwald DS: **The influence of community-based participatory research principles on the likelihood of participation in health research in American Indian communities**. 2007, **17**(1 Suppl 1):S6-14.

37. Sood A, Prasad K, Chhatwani L, Shinozaki E, Cha SS, Loehrer LL, Wahner-Roedler DL: **Patients' attitudes and preferences about participation and recruitment strategies in clinical trials**. 2009, **84**(3):243-247.

38. Thomas B, Stamler LL, Malinowski A: **Collaborating for breast health education and research. A university, industry, and community agency partnership**. 1999, **47**(11):507-511.

39. White MA, Verhoef MJ: **Toward a patient-centered approach: incorporating principles of participatory action research into clinical studies**. 2005, **4**(1):21-24.

40. Abma TA: **Patient participation in health research: research with and for people with spinal cord injuries**. 2005, **15**(10):1310-1328.

41. Abma TA, Broerse JEW: **Patient participation as dialogue: setting research agendas**. 2010, **13**(2):160-173.

42. Agard A, Hermeren G, Herlitz J: **Patients' experiences of intervention trials on the treatment of myocardial infarction: is it time to adjust the informed consent procedure to the patient's capacity?** 2001, **86**(6):632-637.

43. Ard JD, Durant RW, Edwards LC, Svetkey LP: **Perceptions of African-American culture and implications for clinical trial design**. 2005, **15**(2):292-299.

44. Asai A, Ohnishi M, Nishigaki E, Sekimoto M, Fukuhara S, Fukui T: **Focus group interviews examining attitudes toward medical research among the Japanese: a qualitative study**. 2004, **18**(5):448-470.

45. Council AGNHaMR: **A Model Framework for Consumer and Community Participation in Health and Medical Research**. In*.* Canberra 2005.

46. Brody JL, Annett RD, Scherer DG, Turner C, Dalen J: **Enrolling adolescents in asthma research: adolescent, parent, and physician influence in the decision-making process**. 2009, **46**(5):492-497.

47. Campbell M, Copeland B, Tate B: **Taking the standpoint of people with disabilities in research: experiences with participation**. 1998, **12**(2):95-104.

48. Carey MA, Smith MW: **Enhancement of validity through qualitative approaches. Incorporating the patient's perspective**. 1992, **15**(1):107-114.

49. Carey MP, Morrison-Beedy D, Carey KB, Maisto SA, Gordon CM, Pedlow CT: **Psychiatric outpatients report their experiences as participants in a randomized clinical trial**. 2001, **189**(5):299-306.

50. Caron-Flinterman J, Broerse JE, Bunders JF: **The experiential knowledge of patients: A new resource for biomedical research?** 2005, **60**(11):2575-2584.

51. Carr A, Hewlett S, Hughes R, Mitchell H, Ryan S, Carr M, Kirwan J: **Rheumatology outcomes: the patient's perspective**. 2003, **30**(4):880-883.

52. Casarett D, Karlawish J, Sankar P, Hirschman K, Asch DA: **Designing pain research from the patient's perspective: What trial end points are important to patients with chronic pain?** 2001, **2**(4):309-316.

53. Chenoweth L, Kilstoff K: **Facilitating positive changes in community dementia management through participatory action research**. 1998, **4**(3):175-188.

54. Corneli AL, Piwoz EG, Bentley ME, Moses A, Nkhoma JR, Tohill BC, Adair L, Mtimuni B, Ahmed Y, Duerr A *et al*: **Involving communities in the design of clinical trial protocols: the BAN Study in Lilongwe, Malawi**. 2007, **28**(1):59-67.

55. Cotterell P: **Exploring the value of service user involvement in data analysis: 'Our interpretation is about what lies below the surface'**. 2008, **16**(1):5-17.

56. Cox K, Avis M: **Psychosocial aspects of participation in early anticancer drug trials. Report of a pilot study**. 1996, **19**(3):177-186.

57. Cox K: **Enhancing cancer clinical trial management: recommendations from a qualitative study of trial participants' experiences**. 2000, **9**(4):314-322.

58. Curry R: **Vision to reality: using patients' voices to develop and improve services**. 2006, **11**(10):438-445.

59. Daly W: **"Adding their flavour to the mix": involving children and young people in care in research design**. 2009, **62**(4):460-475.

60. Daugherty CK: **Impact of therapeutic research on informed consent and the ethics of clinical trials: a medical oncology perspective**. 1999, **17**(5):1601-1617.

61. Dellson P, Nilbert M, Bendahl PO, Malmstrom P, Carlsson C: **Towards optimised information about clinical trials; identification and validation of key issues in collaboration with cancer patient advocates**. 2011, **20**(4):445-454.

62. Dixon-Woods M, Jackson C, Windridge KC, Kenyon S: **Receiving a summary of the results of a trial: qualitative study of participants' views**. 2006, **332**(7535):206-210.

63. Eng M, Taylor L, Verhoef M, Ernst S, Donnelly B: **Understanding participation in a trial comparing cryotherapy and radiation treatment**. 2005, **12**(2):2607-2613.

64. Featherstone K, Donovan JL: **Random allocation or allocation at random? Patients' perspectives of participation in a randomised controlled trial**. 1998, **317**(7167):1177-1180.

65. Fern E, Kristinsdóttir G: **Young people act as consultants in child-directed research: an action research study in Iceland**. 2011, **16**(3):287-297.

66. Forbes LJL, Nicholls CM, Linsell L, Graham J, Tompkins C, Ramirez AJ: **Involving users in the design of a randomised controlled trial of an intervention to promote early presentation in breast cancer: qualitative study**. 2010, **10**:110.

67. Garber M, Hanusa BH, Switzer GE, Mellors J, Arnold RM: **HIV-infected African Americans are willing to participate in HIV treatment trials**. 2007, **22**(1):17-42.

68. Gittelsohn J, Roache C, Kratzmann M, Reid R, Ogina J, Sharma S: **Participatory research for chronic disease prevention in Inuit communities**. 2010, **34**(4):453-464.

69. Gooberman-Hill R, Horwood J, Calnan M: **Citizens' juries in planning research priorities: process, engagement and outcome**. 2008, **11**(3):272-281.

70. Harper GW, Salina DD: **Building collaborative partnerships to improve community-based HIV prevention research: The University-CBO Collaborative partnership (UCCP) model**. 2000, **19**(1):1-20.

71. Higgins DL, Metzler M: **Implementing community-based participatory research centers in diverse urban settings**. 2001, **78**(3):488-494.

72. Hsu C, Bluespruce J, Sherman K, Cherkin D: **Unanticipated benefits of CAM therapies for back pain: an exploration of patient experiences**. 2010, **16**(2):157-163.

73. Hutchison C: **Phase I trials in cancer patients: participants' perceptions**. 1998, **7**(1):15-22.

74. Irani L, Lin SY, Clipp SL, Alberg AJ, Navas-Acien A: **Involving stakeholders to optimize a study protocol on secondhand tobacco smoke and chronic rhinosinusitis in adults**. 2010, **24**(1):39-44.

75. Jenkins V, Fallowfield L, Cox A: **The preferences of 600 patients for different descriptions of randomisation**. 2005, **92**(5):807-810.

76. Jinks C, Ong BN, Neill TJO: **The Keele community knee pain forum: action research to engage with stakeholders about the prevention of knee pain and disability**. 2009, **10**:85.

77. Karlawish J, Kim SY, Knopman D, Dyck CHv, James BD, Marson D: **The views of Alzheimer disease patients and their study partners on proxy consent for clinical trial enrollment**. 2008, **16**(3):240-247.

78. Karmaliani R, McFarlane J, Asad N, Madhani F, Hirani S, Shehzad S, Zaidi A: **Applying community-based participatory research methods to improve maternal and child health in Karachi, Pakistan**. 2009, **57**(4):204-209.

79. Kelly PJ: **Practical suggestions for community interventions using participatory action research**. 2005, **22**(1):65-73.

80. King G, Servais M, Kertoy M, Specht J, Currie M, Rosenbaum P, Law M, Forchuk C, Chalmers H, Willoughby T: **A measure of community members' perceptions of the impacts of research partnerships in health and social services**. 2009, **32**(3):289-299.

81. Kirwan J, Heiberg T, Hewlett S, Hughes R, Kvien T, Ahlmen M, Boers M, Minnock P, Saag K, Shea B *et al*: **Outcomes from the Patient Perspective Workshop at OMERACT 6**. 2003, **30**(4):868-872.

82. Kirwan JR, Hewlett SE, Heiberg T, Hughes RA, Carr M, Hehir M, Kvien TK, Minnock P, Newman SP, Quest EM *et al*: **Incorporating the patient perspective into outcome assessment in rheumatoid arthritis - progress at OMERACT 7**. 2005, **32**(11):2250-2256.

83. Lammers J, Happell B: **Research involving mental health consumers and carers: a reference group approach**. 2004, **13**(4):262-266.

84. Lavender T, Kingdon C: **Primigravid women's views of being approached to participate in a hypothetical term cephalic trial of planned vaginal birth versus planned cesarean birth**. 2009, **36**(3):213-219.

85. Lindenmeyer A, Hearnshaw H, Sturt J, Ormerod R, Aitchison G: **Assessment of the benefits of user involvement in health research from the Warwick Diabetes Care Research User Group: a qualitative case study**. 2007, **10**(3):268-277.

86. Llewellyn-Thomas HA, Thiel EC, Clark RM: **Patients versus surrogates: whose opinion counts on ethics review panels?** 1989, **37**(3):501-505.

87. MacKinnon S, Stephens S: **Is participation having an impact? Measuring progress in Winnipeg's inner city through the voices of community-based program participants**. 2010, **10**(3):283-300.

88. Manson SM, Garroutte E, Goins RT, Henderson PN: **Access, relevance, and control in the research process: lessons from Indian country**. 2004, **16**(5 Suppl):58S-77S.

89. Martin RE, Murphy K, Chan R, Ramsden VR, Granger-Brown A, Macaulay AC, Kahlon R, Ogilvie G, Hislop TG: **Primary health care: applying the principles within a community-based participatory health research project that began in a Canadian women's prison**. 2009, **16**(4):43-53.

90. Maslin-Prothero S: **Developing user involvement in research**. 2003, **12**(3):412-421.

91. Mastwyk M, Ritchie CW, LoGiudice D, Sullivan KA, Macfarlane S: **Carer impressions of participation in Alzheimer's disease clinical trials: what are their hopes? And is it worth it?** 2002, **14**(1):39-45.

92. McQuiston C, Parrado EA, Martinez AP, Uribe L: **Community-based participatory research with Latino community members: horizonte Latino**. 2005, **21**(4):210-215.

93. Mease P, Arnold LM, Bennett R, Boonen A, Buskila D, Carville S, Chappell A, Choy E, Clauw D, Dadabhoy D *et al*: **Fibromyalgia syndrome**. 2007, **34**(6):1415-1425.

94. Medd JC, Stockler MR, Collins R, Lalak A: **Measuring men's opinions of prostate needle biopsy**. 2005, **75**(8):662-664.

95. Milewa T, Buxton M, Hanney S: **Lay involvement in the public funding of medical research: expertise and counter-expertise in empirical and analytical perspective**. 2008, **18**(3):357-366.

96. Mills N, Donovan JL, Smith M, Jacoby A, Neal DE, Hamdy FC: **Perceptions of equipoise are crucial to trial participation: a qualitative study of men in the ProtecT study**. 2003, **24**(3):272-282.

97. Minkler M, Fadem P, Perry M, Blum K, Moore L, Rogers J: **Ethical dilemmas in participatory action research: a case study from the disability community**. 2002, **29**(1):14-29.

98. Moreno-Black G, Shor-Posner G, Miguez MJ, Burbano X, Mellan SO, Yovanoff P: **"I will miss the study, God bless you all": participation in a nutritional chemoprevention trial**. 2004, **14**(4):469-475.

99. Morin SF, Morfit S, Maiorana A, Aramrattana A, Goicochea P, Mutsambi JM, Robbins JL, Richards TA: **Building community partnerships: case studies of Community Advisory Boards at research sites in Peru, Zimbabwe, and Thailand**. 2008, **5**(2):147-156.

100. Murad MH, Shah ND, Houten HKV, Ziegenfuss JY, Deming JR, Beebe TJ, Smith SA, Guyatt GH, Montori VM: **Individuals with diabetes preferred that future trials use patient-important outcomes and provide pragmatic inferences**. 2011, **64**(7):743-748.

101. Nair K, Willison D, Holbrook A, Keshavjee K: **Patients' consent preferences regarding the use of their health information for research purposes: a qualitative study**. 2004, **9**(1):22-27.

102. Ntshanga SP, Ngcobo PS, Mabaso ML: **Establishment of a Community Advisory Board (CAB) for tuberculosis control and research in the Inanda, Ntuzuma and KwaMashu (INK) area of KwaZulu-Natal, South Africa**. 2010, **95**(2-3):211-215.

103. Ong BN, Hooper H: **Involving users in low back pain research**. 2003, **6**(4):332-341.

104. Paul CL, Sanson-Fisher R, Douglas HE, Clinton-McHarg T, Williamson A, Barker D: **Cutting the research pie: a value-weighting approach to explore perceptions about psychosocial research priorities for adults with haematological cancers**. *European Journal of Cancer Care* 2011, **20**(3):345-353.

105. Plumb M, Collins N, Cordeiro JN, Kavanaugh-Lynch M: **Assessing process and outcomes: evaluating community-based participatory research**. 2008, **2**(2):85-86, 87-97.

106. Read K, Fernandez CV, Gao J, Strahlendorf C, Moghrabi A, Pentz RD, Barfield RC, Baker JN, Santor D, Weijer C *et al*: **Decision-making by adolescents and parents of children with cancer regarding health research participation**. 2009, **124**(3):959-965.

107. Reddy P, Buchanan D, Sifunda S, James S, Naidoo N: **The role of community advisory boards in health research: Divergent views in the South African experience**. 2010, **7**(3):2-8.

108. Redwood D, Lanier A, Kemberling M, Klejka J, Sylvester I, Lundgren K: **Community-based participatory research in a large cohort study of chronic diseases among Alaska native adults**. 2010, **4**(4):325-330.

109. Richards L, Kennedy PH, Krulewitch CJ, Wingrove B, Katz K, Wesley B, Feinson C, Herman A: **Achieving success in poor urban minority community-based research: strategies for implementing community-based research within an urban minority**. 2002, **3**(3):410-420.

110. Roberts LW, Warner TD, Brody JL: **Perspectives of patients with schizophrenia and psychiatrists regarding ethically important aspects of research participation**. 2000, **157**(1):67-74.

111. Roberts LW, Warner TD, Brody JL, Roberts B, Lauriello J, Lyketsos C: **Patient and psychiatrist ratings of hypothetical schizophrenia research protocols: assessment of harm potential and factors influencing participation decisions**. 2002, **159**(4):573-584.

112. Roberts LW, Warner TD, Anderson CT, Smithpeter MV, Rogers MK: **Schizophrenia research participants' responses to protocol safeguards: recruitment, consent, and debriefing**. 2004, **67**(2-3):283-291.

113. Roe KM, Minkler M, Saunders FF: **Combining research, advocacy, and education: the methods of the Grandparent Caregiver Study**. 1995, **22**(4):458-475.

114. Rogers ES, Danley KS, Anthony WA, Martin R, Walsh D: **The residential needs and preferences of persons with serious mental illness: a comparison of consumers and family members**. *Journal of Mental Health Administration* 1994, **21**(1):42-51.

115. Rosen C, Grossman LS, Sharma RP, Bell CC, Mullner R, Dove HW: **Subjective evaluations of research participation by persons with mental illness**. 2007, **195**(5):430-435.

116. Ross F, Donovan S, Brearley S, Victor C, Cottee M, Crowther P, Clark E: **Involving older people in research: methodological issues**. 2005, **13**(3):268-275.

117. Saurbrey N, Jensen J, Rasmussen PE, Gjorup T, Guldager H, Riis P: **Danish patients' attitudes to scientific-ethical questions. An interview study focusing therapeutic trials**. 1984, **215**(2):99-104.

118. Savage CL, Xu Y, Lee R, Rose BL, Kappesser M, Anthony JS: **A case study in the use of community-based participatory research in public health nursing**. 2006, **23**(5):472-478.

119. Sayers J: **Clinical trial registries: a survey of patient advocate group perceptions**. 2009, **43**(2):195-200.

120. Serrano-Aguilar P, Trujillo-Martin MM, Ramos-Goni JM, Mahtani-Chugani V, Perestelo-Perez L, Paz MP-dl: **Patient involvement in health research: a contribution to a systematic review on the effectiveness of treatments for degenerative ataxias**. 2009, **69**(6):920-925.

121. Shilling V, Williamson PR, Hickey H, Sowden E, Beresford MW, Smyth RL, Young B: **Communication about children's clinical trials as observed and experienced: qualitative study of parents and practitioners**. 2011, **6**(7):e21604.

122. Slomka J, Ratliff EA, McCurdy SA, Timpson S, Williams ML: **Decisions to participate in research: Views of underserved minority drug users with or at risk for HIV**. 2008, **20**(10):1224-1232.

123. Smith YR, Johnson AM, Newman LA, Greene A, Johnson TRB, Rogers JL: **Perceptions of clinical research participation among African American women**. 2007, **16**(3):423-428.

124. Snowdon C, Elbourne D, Garcia J: **"It was a snap decision": Parental and professional perspectives on the speed of decisions about participation in perinatal randomised controlled trials**. 2006, **62**(9):2279-2290.

125. Snowdon C, Garcia J, Elbourne D: **Making sense of randomization; responses of parents of critically ill babies to random allocation of treatment in a clinical trial**. *Social Science & Medicine* 1997, **45**(9):1337-1355.

126. Staniszewska S, Jones N, Newburn M, Marshall S: **User involvement in the development of a research bid: barriers, enablers and impacts**. 2007, **10**(2):173-183.

127. Staniszewska S, Brett J, Mockford C, Barber R: **The GRIPP checklist: strengthening the quality of patient and public involvement reporting in research**. *International Journal of Technology Assessment in Health Care* 2011, **27**(4):391-399.

128. Stewart P, Shibasaki S, Anderson I, Pyett P, Dunbar T, Devitt J: **Aboriginal and Torres Strait Islander participation in ethical review of health research**. 2006, **30**(3):291-292.

129. Stewart MK, Colley D, Huff A, Felix H, Shelby B, Strickland E, Redmond P, Evans M, Baker B, Stephens G *et al*: **Participatory development and implementation of a community research workshop: experiences from a community-based participatory research partnership**. 2009, **3**(2):165-178.

130. Stirman SW, Goldstein LA, Wrenn G, Barrett M, Gibbons MBC, Casiano D, Thompson D, Green PP, Heintz L, Barber JP *et al*: **Developing research and recruitment while fostering stakeholder engagement in a National Institutes of Mental Health-funded Interventions and Practice Research Infrastructure Programs grant for depression**. 2010, **4**(4):299-303.

131. Sugarman J, Kass NE, Goodman SN, Perentesis P, Fernandes P, Faden RR: **What patients say about medical research**. 1998, **20**(4):1-7.

132. Sullivan M, Bhuyan R, Senturia K, Shiu-Thornton S, Ciske S: **Participatory action research in practice: a case study in addressing domestic violence in nine cultural communities**. 2005, **20**(8):977-995.

133. Thurston WE, MacKean G, Vollman A, Casebeer A, Weber M, Maloff B, Bader J: **Public participation in regional health policy: A theoretical framework**. 2005, **73**(3):237-252.

134. Timotijevic L, Raats MM: **Evaluation of two methods of deliberative participation of older people in food-policy development**. 2007, **82**(3):302-319.

135. Tischler V, Silva KD, Cheetham A, Goring M, Calton T: **Involving Patients in Research: the Challenge of Patient-Centredness**. 2010, **56**(6):623-633.

136. Tobin M, Chen L, Leathley C: **Consumer participation in mental health services: who wants it and why?** 2002, **25**(3):91-100.

137. Olphen JV, Ottoson J, Green L, Barlow J, Koblick K, Hiatt R: **Evaluation of a partnership approach to translating research on breast cancer and the environment**. 2009, **3**(3):213-226.

138. Staa Av, Jedeloo S, Latour JM, Trappenburg MJ: **Exciting but exhausting: experiences with participatory research with chronically ill adolescents**. 2010, **13**(1):95-107.

139. Wersch Av, Eccles M: **Involvement of consumers in the development of evidence based clinical guidelines: practical experiences from the North of England evidence based guideline development programme**. 2001, **10**(1):10-16.

140. White CD, Hardy JR, Gilshenan KS, Charles MA, Pinkerton CR: **Randomised controlled trials of palliative care - a survey of the views of advanced cancer patients and their relatives**. 2008, **44**(13):1820-1828.

141. Wright JG, Young NL: **The patient-specific index: asking patients what they want**. 1997, **79**(7):974-983.

142. Zullino D, Conus P, Borgeat F, Bonsack C: **Readiness to participate in psychiatric research**. 2003, **48**(7):480-484.
